# Dose threshold for radiation induced fetal programming in a mouse model at 4 months of age: Hepatic expression of genes and proteins involved in glucose metabolism and glucose uptake in brown adipose tissue

**DOI:** 10.1371/journal.pone.0231650

**Published:** 2020-04-21

**Authors:** Caitlund Q. Davidson, Sujeenthar Tharmalingam, Sarah Niccoli, Ashley Nemec-Bakk, Sandhya Khurana, Alyssa Murray, T. C. Tai, Douglas R. Boreham, Neelam Khaper, Simon J. Lees

**Affiliations:** 1 Department of Biology, Lakehead University, Thunder Bay, Ontario, Canada; 2 Division of Medical Sciences, Northern Ontario School of Medicine, Laurentian University, Sudbury, Ontario, Canada; 3 Department of Medical Physics and Applied Radiation Sciences, McMaster University, Hamilton, Ontario, Canada; 4 Division of Medical Sciences, Northern Ontario School of Medicine, Lakehead University, Thunder Bay, Ontario, Canada; University of Southampton, UNITED KINGDOM

## Abstract

Exposure to ionizing radiation contributing to negative health outcomes is a widespread concern. However, the impact of low dose and sub-lethal dose radiation (SLDR) exposures remain contentious, particularly in pregnant women who represent a vulnerable group. The fetal programming hypothesis states that an adverse *in utero* environment or stress during development of an embryo or fetus can result in permanent physiologic changes often resulting in progressive metabolic dysfunction with age. To assess changes in gene expression profiles of glucose/insulin signaling and lipid metabolism caused by radiation exposure *in utero*, pregnant C57Bl/6J mice were irradiated using a dose response ranging from low dose to SLDR and compared to a Sham-irradiated group. mRNA expression analysis in 16 week old offspring (n = 84) revealed that genes involved in metabolic function including glucose metabolism, insulin signaling and lipid metabolism were unaffected by prenatal radiation exposures up to 300 mGy. However, female offspring of dams exposed to 1000 mGy had upregulated expression of genes contributing to insulin resistance and gluconeogenesis. In a second cohort of mice, the effects of SLDR on fetal programming of hepatic SOCS3 and PEPCK protein expression were assessed. 4 month old female offspring of dams irradiated at 1000 mGy had: 1) increased liver weights, 2) increased hepatic expression of proteins involved in glucose metabolism and 3) increased ^18^F-fluorodeoxyglucose (FDG) uptake in interscapular brown adipose tissue (IBAT) measured by positron emission tomography (PET) (n = 25). The results of this study indicate that prenatal radiation exposure does not affect metabolic function up to 300 mGy and 1000 mGy may be a threshold dose for sex-specific alterations in glucose uptake and hepatic gene and protein expression of SOCS3, PEPCK, PPARGC1A and PPARGC1B. These findings suggest that SLDR doses alter glucose uptake in IBAT and hepatic gene and protein expression of offspring and these changes may progress with age.

## Introduction

Exposure to ionizing radiation and the possibility of radiation contributing to negative health outcomes is a widespread concern among the public, scientific community, and workers in the nuclear energy industry and diagnostic imaging. Pregnant women represent a vulnerable group and are sometimes exposed to radiation during diagnostic imaging if the woman is not aware of the pregnancy or in emergency situations. Effects of exposure on the fetus are dependent on gestational age and absorbed radiation dose. An adverse *in utero* environment or stress during development of an embryo or fetus can result in permanent physiologic changes, known as fetal programming [1]. These changes often result in progressive metabolic dysfunction with age. Studies have shown that changes in the offspring from fetal programming appear at 3 months of age and persist to 18 months of age [2–4]. The effects of adverse effects during pregnancy can even have a negative impact on the fetus. Fetal programming models have shown low maternal dietary protein impacts body composition, body weight, metabolism, and hormone balance while glucocorticoid exposure can affect fetal growth, blood pressure and glucose metabolism [5, 6]. There are several established models of fetal programming that induce *in utero* stress including maternal undernutrition, protein-restricted diets, maternal obesity, prenatal hypoxia, and exposure to stress hormones [7–15]. These models all result in similar outcomes with the offspring demonstrating alterations in birth weight followed by weight normalization within the first few months. Intrauterine growth restriction and low birth weight seem to predispose offspring to insulin resistance and impaired glucose metabolism [7–9]. Glucocorticoid exposure, maternal obesity, maternal undernutrition or diet restriction predispose animals to alterations in metabolism and insulin resistance [10–13]. Prenatal hypoxia contributes to cardiovascular disease development in adulthood [14]. Increased adiposity in offspring is seen in models of maternal obesity [15]. Overall, a poor maternal state leads to placental insufficiency and negative health outcomes of the offspring.

Many of the putative effects of radiation on the fetus have been studied in high dose animal studies and from atomic bomb exposure [16, 17]. Sreetharan et al. reviewed the effects on postnatal development when exposed to radiation at different phases pregnancy [18]. During the first trimester, high doses of radiation exposure has an all-or-nothing effect where radiation can be lethal or have no effects at all to the embryo. If the embryo survives, it is likely to develop fully with a low risk for congenital abnormalities. It is generally accepted that exposures to low doses during the first two weeks of pregnancy may cause damage that can be compensated for or repaired. Low doses are detrimental during organogenesis. There are increased risks of malformations, growth restriction, and behavioural or motor skill deficits discussed by De Santis et al. 2007 [19]. Radiation risks are somewhat less in the second trimester compared to the first semester but more diverse [20]. There is still a risk of mortality but there is the additional risk of the offspring having intrauterine growth restriction and intellectual disabilities [17]. The third trimester, or late gestation, is a critical stage where cells can be influenced by changes in the in-utero environment resulting in adaptations in cellular function [21–23]. Fetal programming changes are more diverse in the second and third trimesters of pregnancy compared to the first trimester based on the animal models reviewed by Sreetharan et al. 2017. Gestation day 15 (GD15) was chosen for this model because previous fetal programming studies have shown that exposure during the third trimester, usually gestational days 15 to19, induce observable changes in offspring [24–27]. Radiation exposure to 1000 mGy in the third trimester of pregnancy in humans, has resulted in behavioural, social, and locomotor changes in the offspring in adulthood as well as the appearance of microcephaly [24, 25, 28]. However, little is known about the effects of low dose radiation (LDR) and sub-lethal dose radiation (SLDR) on fetal programming of hepatic genes and proteins involved in glucose metabolism and glucose uptake.

United Nations Scientific Committee on the Effects of Atomic Radiation classifies LDR as any radiation dose below 100 mGy [29]. High dose radiation varies significantly ranging between 2000 mGy and 15 000 mGy [30–33]. High doses are usually accumulated during multiple exposures rather than a one-time whole-body exposure. It is also important to note that there are major differences in mouse strain radiation sensitivity. Radiation sensitivity is referenced as LD_50:30_ which is the dose of whole-body radiation that is lethal to 50% of the target population by 30 days after exposure. Grahn and Hamilton 1956, demonstrated that the C57Bl/6 mice had an LD_50:30_ of 6300 ± 40 mGy and considered this strain to be radioresistant [34]. In comparison, BALB/c mice are considered radiosensitive with an LD_50:30_ of 5000 ± 60 mGy. In humans, the LD_50:30_ is approximately 4500 mGy [35]. With this knowledge, the amount of radiation can be adjusted to be comparable to the doses and physiological risks in humans.

The purpose of this study was to investigate the effects of a dose response of ionizing radiation during the third trimester of pregnancy on liver mRNA and protein expression involved in glucose metabolism and glucose uptake in IBAT in the offspring of irradiated dams at 16 weeks of age. For the present study, a radiation dose response of 0 (Sham), 5, 10, 50, 300, or 1000 mGy was used. 5, 10 and 50 mGy represent low dose radiation, while 300 and 1000 mGy are considered a SLDR. We hypothesized that SLDR exposure would cause alterations in the expression of proteins involved in glucose metabolism potentially leading to insulin resistance and increased gluconeogenesis, while glucose uptake in IBAT would increase to compensate for impaired whole-body glucose metabolism.

## Materials and methods

Institutional animal care approval was received from Lakehead University Animal Care Committee (AUP#1465023) and the Animal Research Ethics Board at McMaster University (AUP#15-11-26). Canadian Council on Animal Care (CCAC) standards were followed for the welfare of the animals in the present study.

### Breeding

Male and female C57Bl/6J wildtype mice were obtained from Jackson Laboratory (Bar Harbor, Maine, USA) aged 7–8 weeks. Animals were maintained on a 12:12 hour light:dark cycle and allowed food and water *ad-libitum*. Housing temperature was controlled, at 20–22°C. The mice were given one week to acclimate without disruption prior to breeding putting the mice at 8–9 weeks of age at the time of breeding. After weaning, housing temperature was maintained within the CCAC standards (18–22°C). Females were housed 5 per cage from arrival until breeding. Males were individually housed for the duration of the study. Female mice were moved to a male cage (2 females:1 male) and allowed to breed overnight. The following morning, females were removed from the male cages and individually housed. Vaginal plugs were used to determine the first day of gestation.

Litter sizes ranged between 3–9 offspring. Culling was not used to ensure a certain number or equal sex as it is controversial whether it is beneficial [36, 37]. All pups were maintained after delivery. Early handling of the offspring and cross-fostering were not performed to avoid additional stress and changes in behaviour [38, 39]. A maximum of two pups from a single mother was used in this study to control maternal effects. The pups not assigned to this were used for a different study at a different location. Only the number needed for the study were sent to the study location. Gene expression results were obtained using a total of 42 female and 42 male mice at 16 week of age. 25 offspring (17 males, 18 females) were used for PET imaging and western blot analysis of the liver and BAT.

The mice that were anesthetized for the 10 minute imaging were kept warm. Upon completion of study procedures, the mice were fasted for 5 hours prior to being euthanized at 4.5 months of age ± 1 week. A short 5 hour morning fast in mice is sufficient to assess metabolism as prolonged fasting can increase insulin-stimulated glucose utilization [40–42]. An overdose of inhaled isoflurane anesthetic was used for euthanasia in combination with exsanguination, which is in line with the recommendations of the CCAC.

### Radiation

Radiation treatments were done at the Taylor Source facility at McMaster University. Pregnant females were irradiated a single time at 0 (Sham), 5, 10, 50, 300, or 1000 mGy on day 15 of gestation using ^137^Cs gamma radiation (662 keV energy) (Taylor Radiobiology Source). The mice were placed under the source for 20 minutes prior to irradiation in their home cage. Sham-irradiated animals were placed under the shielded source for 20 minutes and were then moved to the control room for the duration of the irradiation. Access to food and water were restricted for the period of irradiation (Sham-irradiated animals included). Radiation was delivered at a dose rate of 10 mGy/min measured using thermoluminescent dosimeters (Mirion Technologies, Irvine, California, USA) placed in the bedding of an empty animal cage with the lid on.

### RNA extraction and cDNA synthesis

In the first cohort of animals, RNA was extracted. Here, the entire liver was homogenized in TRI reagent (Sigma Life Science, Missouri, USA) using a Tissuelyser (Qiagen, Ontario, Canada) for 2 cycles at 30 Hz for 2 min. Total RNA was extracted from the TRI Reagent (Sigma Life Science, Missouri, USA) homogenate according to manufacturer’s instructions. RNA concentration and purity (260/280 absorbance) were determined using ND-1000 spectrophotometer. Contaminating genomic DNA was removed from the RNA samples using the DNAse kit (Sigma Life Science, Missouri, USA) according to manufacturer’s instructions. The DNAse treated RNA samples were reverse transcribed using random primers (Sigma Life Science, Missouri, USA), oligo dT (VWR), and M-MLV reverse transcriptase (Promega) according to manufacturer’s instructions to obtain complementary DNA (cDNA).

### Real-time quantitative polymerase chain reaction (RT-qPCR)

RT-qPCR reactions were performed using QuantStudio5 (ThermoFisher, Massachusetts, USA) to obtain threshold cycle (Ct) values. Each reaction was performed in 15 μL volumes containing 1 × SensiFast qPCR FastMix (FroggaBio, Ontario, Canada), 600 nM forward/reverse primers and 6 ng cDNA. The cycling conditions were as follows: (1) 95 °C for 2 minutes, (2) 95 °C for 10 seconds, (3) 58 °C for 15 seconds, (4) 72 °C for 20 seconds, (5) plate read and data collection, and (6) steps 2 to 5 repeated for 40 cycles. DNA amplification specificity was determined by performing DNA melt curve analysis at the end of each qPCR run.

Primer-BLAST (NCBI, Maryland, USA) software was utilized to design forward and reverse primer pair sequences for genes of interest (S1 Table). All primer pairs were subjected to stringent validation tests: primers with amplification efficiency between 90% to 110% (efficiency = [10^(−1/slope)^-1; slope determined by plotting Ct values against cDNA serial dilutions), and R^2^ value greater than 0.99 were considered validated and acceptable for qPCR analysis. All samples were normalized to two independent control housekeeping genes (β-actin and rpl29). The relative mRNA transcript level of each gene was reported according to the ΔΔ*C*_*T*_ method as mRNA fold increase [43].

### PET imaging

In a second cohort of animals, glucose uptake in interscapular brown adipose tissue (IBAT) was assessed using ^8^F-fluorodeoxyglucose (FDG) obtained from the cyclotron (Thunder Bay Regional Health Research Institute, Thunder Bay, ON, Canada) on experiment days. A concentration of approximately 300 μCi/ml diluted with sterile saline was made. A mouse was anesthetized with 1.5% isoflurane anesthetic for five minutes inside a vapour induction chamber. An intraperitoneal injection of the appropriate volume at a dose of approximately 20 μCi was given followed by a 55-minute uptake period where the mouse was returned to its cage. After the uptake period, the mouse was anaesthetized again and imaged in a G4 PET/X-ray scanner (Sofie Biosciences, Culver City, California, USA). A 10-minute acquisition was performed followed by an X-ray. The mouse was then returned to its cage to recover. Images were analyzed using VivoQuant^™^(Version 1.23, Invicro, Boston).

### Sample preparation and western blot analysis

One week after PET image acquisition, mice were anesthetized under 3% isoflurane anesthetic and the hearts removed. IBAT and livers were immediately frozen on dry ice. Frozen liver tissue was pulverized into powder using a mortar and pestle kept cold with liquid nitrogen and kept on dry ice. 100 mg of frozen liver powder was homogenized in ice-cold lysis buffer (25mmol/L Tris pH = 7.5, 150 mmol/L NaCl, 1 mmol/L EDTA, 1% Triton-X 100) with an addition of sodium fluoride, sodium orthovanadate and protease inhibitor (Sigma Life Science, Missouri, USA). Frozen IBAT (whole) was homogenized in the same buffer but without sodium orthovanadate. Homogenization was completed using the Qiagen TissueLyser. Samples were centrifuged at 16000 g for 10 minutes at 4°C and supernatants were collected. From the IBAT samples only the intermediate supernatant layer was collected to avoid the fatty top layer. IBAT supernatant was centrifuged a second time and intermediate layer collected again to clear any remaining lipids. Prior to performing western blots, protein assays (Pierce^™^ BCA Protein Assay Kit, Thermo Scientific, Illinois, USA) were completed to determine protein content for western blot analysis and for sample normalization.

Protein expression was determined using western blots. Polyacrylamide gels were prepared at a 15% concentration for SOCS3 isolation and 10% concentration for PEPCK, UCP1, phosphorylated Akt (pAkt), Akt, phosphorylated GSK3β (pGSK3β), and GSK3β isolation. Samples were prepared using 4X Laemmli buffer with dithiothreitol (Fisher Scientific, New Jersey, USA) and distilled water. Each western blot sample was boiled at 100°C for 5 minutes and then placed on ice until loaded into the gel. 5μl of molecular ladder (BLUelf prestained protein ladder, FroggaBio, Ontario, Canada) was added to the first well. Samples were loaded alternating treatment groups. Male and female samples were run on different gels. Several samples were repeated across gels as loading controls. All gels were run for 1 hour at 200 volts. Each gel was transferred to a nitrocellulose membrane using a wet transfer method.

The membranes were pre-incubated for 1 hour in 25ml each of blocking solution which consisted of 5% powdered milk in 1XTBST (TBST: Tris Base (Fisher Scientific, New Jersey, USA) + Tris Hydrochloride (Fisher Scientific, New Jersey, USA) + Tween 20 (Bio Rad, California, USA) to reduce non-specific protein binding. Target proteins were detected with primary antibodies: SOCS3 (Cell Signaling Technology, Massachusetts, USA), anti-PCK1 (Abcam, Massachusetts, USA), UCP1 (D9D6X) Rabbit mAb (Cell Signaling Technology), Phospho-Akt (Ser473) (D9E) XP^®^ Rabbit mAb (Cell Signaling Technology, Massachusetts, USA), Akt (pan) (C67E7) (Cell Signaling Technology, Massachusetts, USA), Phospho-GSK-3β (Ser9) (Cell Signaling Technology, Massachusetts, USA), GSK-3β XP^®^ Rabbit mAb (Cell Signaling Technology, Massachusetts, USA). All primary antibodies were used at a dilution of 1:1000 except Akt and p-Akt which were 1:500. Primary antibody incubations were performed overnight at 4°C.

After incubation, the membranes were incubated in Pierce antibody goat-anti rabbit IgG (H + L) (Thermo Scientific, Illinois, USA) for 1 hour at room temperature. ChemiDoc XRS by Bio-Rad (California, USA) was used to visualize the bands. SuperSignal West Pico Chemiluminescent Substrate was used for detection of SOCS3 and pAkt bands. For all other targets, immunoreactive complexes were detected with enhanced Chemiluminescence. Quantification of the visualized bands was carried out by ImageJ software. Ponceau S (Fisher Scientific, New Jersey, USA) staining was chosen instead of other loading controls because standard housekeeping proteins (e.g., GAPDH and β-actin) can be affected by different cellular process and may not accurately reflect total protein loads [44–47]. Mortiz reviewed the benefits to total protein staining compared to housekeeping proteins and concluded that total protein staining is the better loading control [45]. Housekeeping proteins have several drawbacks due to technical and biological variations. Ponceau S staining is fast (5–20 min), reversible and does not adversely affect subsequent analytical techniques [46]. Ponceau S was prepared using 0.5g of Ponceau S (Fisher Scientific, New Jersey, USA), 25ml of acetic acid and distilled water. The bottle containing the Ponceau S was stored in the dark as it is light sensitive. Enough Ponceau S was added to cover each membrane and left for 5 minutes. The membranes were rinsed with distilled water and imaged by being placed directly on a computer scanner. This step was done after the overnight transfer and before the blocking.

### Triglyceride assay

Blood was collected from the second cohort of animals during dissection and stored in 10% EDTA on ice. Within two hours, the blood was centrifuged at 3000 g for 10 minutes at 4°C. The supernatant (plasma) was collected and stored at -30°C until analysis. Frozen liver powder was weighed out and homogenized in ice-cold lysis buffer (5% Igepal CA-630, MP Biomedicals, Ohio, USA) with addition of Protease Inhibitor Cocktail (Sigma Life Science, Missouri, USA). Homogenization was completed using the Qiagen TissueLyser (Ontario, Canada). Samples were then centrifuged at 16000 g for 10 minutes at 4°C, supernatants were collected. Protein assays were completed (Pierce^™^ BCA Protein Assay Kit, Thermo Scientific, Illinois, USA) to determine protein content for normalization. Triglyceride assays were completed to determine hepatic and plasma triglyceride content (Triglyceride Colorimetric Assay Kit, Cayman Chemical Company, Michigan, USA). NP-40 from the kit was replaced with a chemically indistinguishable substitute, 5% Igepal CA-630 (MP Biomedicals, Ohio, USA).

### Statistical analysis

All data was presented as means ± SEM. Comparisons between treatment groups were done using two-tailed Student’s *t*-tests with Microsoft Excel or one-way ANOVA followed by Tukey’s post-hoc analysis with SPSS 18 (IBM) software package. A resulting *p*-value of less than or equal to 0.05 was considered statistically significant.

## Results

### Identification of radiation threshold dose for development of altered metabolic health outcomes

An adverse *in utero* environment has been linked to offspring which develop metabolic syndrome diseases in adulthood. In this study, we aimed to identify the metabolic programming effects of single radiation exposure during gestation. In order to identify dose threshold effects, pregnant C57Bl/6J mice were exposed to one of the following radiation doses at GD15: 0 (Sham), 5, 10, 50, 300, or 1000 mGy. Here, doses 5, 10 and 50 mGy resemble radiation doses achieved during diagnostic imaging and are considered LDR, while 300 and 1000 mGy are considered SLDR and may represent relevant doses for the translation of mouse model data to the potential effects in humans.

Negative health outcomes associated with *in utero* insults includes impaired glucose/lipid metabolism, insulin resistance, and altered energy production. In order to identify the metabolic systems altered due to *in utero* radiation exposure, the liver from 4-month old offspring were analyzed using a panel of genes targeting the following systems: glucose metabolism/insulin signaling (genes: PPARGC1A, PPARGC1B, SOCS3, PEPCK, IRS1, GSK, GLUT2) and lipid metabolism (genes: LXRα, LXRβm SREBP-1c, ACACA, FASN, SCD1). The RT-qPCR results from this screen are outlined in S2 Table (female) and S3 Table (male) represented as mRNA expression fold change relative to Sham for all dose groups. The RT-qPCR gene array analysis indicated that mRNA expression of genes involved in metabolic function are unaffected from prenatal radiation exposures up to 300 mGy. This was shown by lack of gene expression changes compared to Sham across a variety of molecular pathways including lipid metabolism and insulin/glucose metabolism.

However, numerous genes were statistically dysregulated at 1000 mGy prenatal radiation exposure (Table 1). Most prominently, female offspring from 1000 mGy dams showed increased expression of genes which contribute to insulin resistance and gluconeogenesis (PPARG1a/b, SOCS3 and PEPCK) at 4 months of age, while genes involved in glucose metabolism were unaffected (GSK, IRS1, GLUT2).

**Table 1:** Liver mRNA expression of 1000 mGy *in utero* exposed offspring.

SYSTEM	GENE	FEMALE	MALE
**Glucose/Insulin Signaling**	**PPARGC1A**	**1.70 ± 0.34**	0.98 ± 0.23
	**PPARGC1B**	**1.60 ± 0.22**	1.26 ± 0.22
	**SOCS3**	**2.01 ± 0.40**	**2.06 ± 0.76**
	**PEPCK**	**2.03 ± 0.39**	1.12 ± 0.16
	**IRS1**	0.92 ± 0.07	1.10 ± 0.11
	**GSK**	0.70 ± 0.11	0.89 ± 0.12
	**GLUT2**	1.27 ± 0.08	1.30 ± 0.25
**Lipid Metabolism**	**LXRα**	1.11 ± 0.06	1.14 ± 0.06
	**LXRβ**	1.25 ± 0.08	1.63 ± 0.31
	**SREBP-1c**	0.84 ± 0.18	0.78 ± 0.11
	**ACACA**	1.02 ± 0.10	1.00 ± 0.14
	**FASN**	0.99 ± 0.22	**0.54 ± 0.14**
	**SCD1**	0.77 ± 0.12	0.90 ± 0.30

Expression represented as fold change (± SEM) relative to Sham. ANOVA: statistical significance (*p* < 0.05) compared to Sham are represented as bolded red text (upregulated) or blue text (downregulated) (n = 7–8 per group).

Interestingly, prenatal radiation exposure did not affect the majority of genes involved in fatty acid biosynthesis at all dose ranges in both females and males. However, males exposed to prenatal 1000 mGy demonstrated reduced FASN (fatty acid synthase) gene expression compared to Sham. Taken together, the gene expression study revealed that radiation does not affect metabolic functions mediated by the liver up to 300 mGy, and 1000 mGy may be a threshold dose for sex-specific metabolic health outcomes. In order to address this, 1000 mGy exposed animals were chosen for further analysis.

### Anthropometric results

In a separate cohort of animals, pregnant dams were exposed to either 0 (Sham) or 1000 mGy of radiation at GD15. Female offspring body weight at 4 months of age exhibited a nonsignificant 6% difference between the 1000 mGy group and the Sham group (*p* = 0.07) (Table 2). There was no difference in body weight in the male offspring caused by maternal exposure to 1000 mGy, compared to the Sham group. It is possible that catch-up growth occurred over 4 months and any differences that may have existed at birth no longer existed.

**Table 2:** Tissue weights from offspring of irradiated and Sham-irradiated dams.

	Male		Female	
	Sham	1000mGy	Sham	1000mGy
Body Weight (g)	27.80 ± 1.11	27.66 ± 0.87	21.14 ± 0.58	19.96 ± 0.23
Liver Weight (mg)	1262.61 ± 116.40	1170.17 ± 63.94	809.19 ± 63.33	959.42 ± 30.28^a^
IBAT Weight (mg)	86.51 ± 8.24	80.09 ± 4.49	50.13 ± 2.53	46.93 ± 2.24
Liver/Body Weight (mg/g)	46.60 ± 3.47	42.30 ± 1.98	38.16 ± 2.64	48.01 ± 1.07^a^
IBAT/Body Weight (mg/g)	2.92 ± 0.24	2.90 ± 0.15	2.36 ± 0.07	2.35 ± 0.10

Measurements were taken at 4 months of age. Tissue weights were taken prior to freezing. Data are presented as means ± SEM. n = 7–10 per group. Two-tailed t-test: Statistical significance between Sham and 1000 mGy groups is shown by * *p* ≤ 0.05.

Tissue weights were collected for liver and IBAT (Table 2). Tissue weights were compared to body weight for each mouse to determine if there was any difference in tissue weight between treatment groups. There was no difference in the IBAT to body weight ratio in females or males with treatment. Liver weight was significantly higher in female offspring from 1000 mGy dams but did not change significantly in the males.

### Proteins involved in glucose metabolism and triglyceride content in the liver

**Fig 1:**
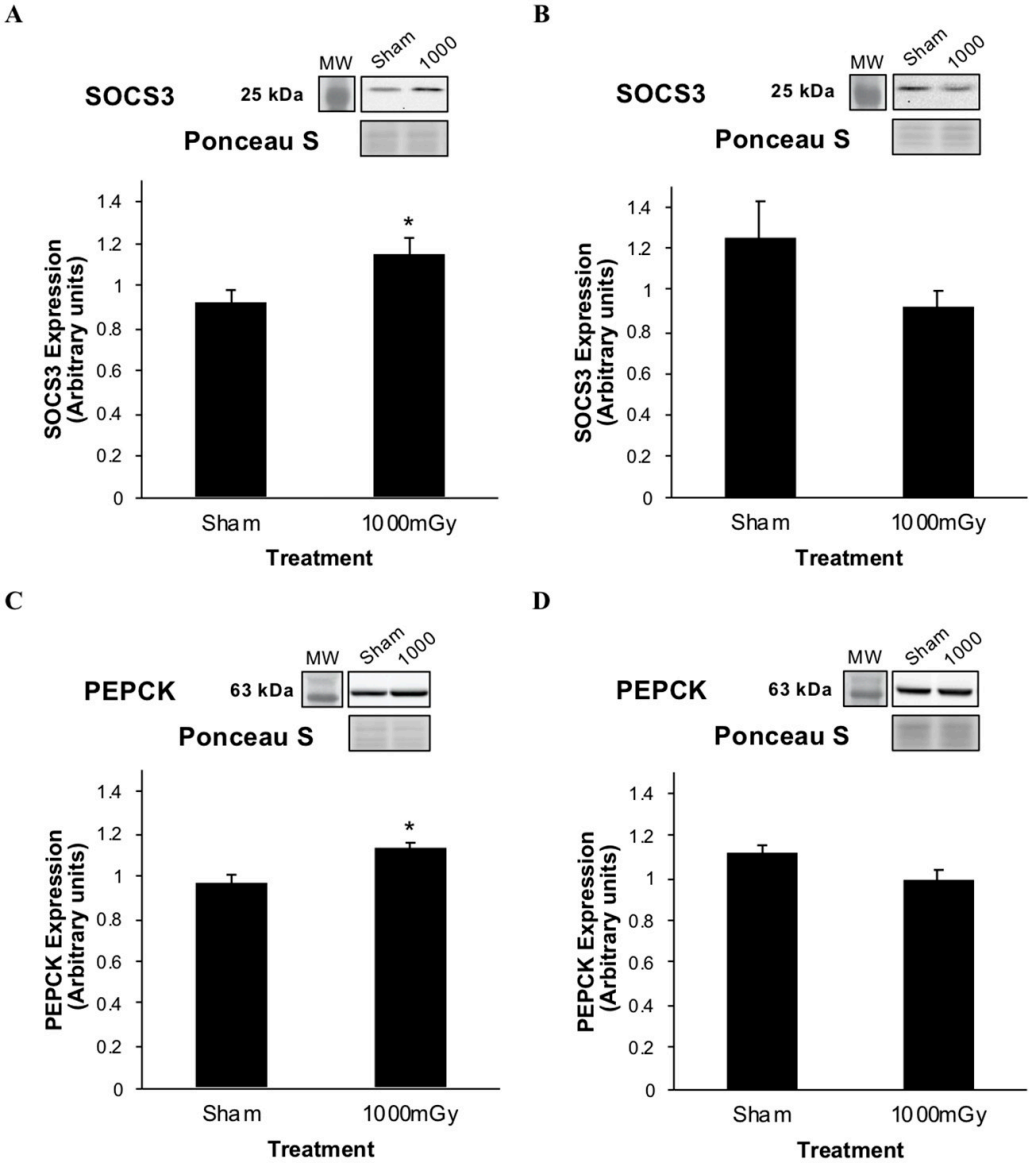
Liver SOCS3 and PEPCK protein expression from offspring of 1000 mGy irradiated and Sham-irradiated dams at 4 months of age. (A) Female offspring liver SOCS3 protein expression. (B) Male offspring liver SOCS3 protein expression. (C) Female offspring liver PEPCK protein expression. (D) Male offspring liver PEPCK protein expression. Results were normalized to loading controls. Ponceau S stains are shown as markers of equal protein loading. Two-tailed t-test: Statistical significance between Sham and 1000 mGy groups is shown by * *p* ≤ 0.05. Data are presented as mean ± SEM. n = 7–10 per group.

The female offspring of 1000 mGy irradiated dams had 20% higher SOCS3 protein expression, compared to offspring from Sham-irradiated dams (Fig 1A). However, in males, there was a non-significant 27% lower SOCS3 protein expression in 1000 mGy, compared to Sham (Fig 1B). Interestingly, these data indicate an opposite effect of irradiation in the female and male offspring. Similarly, the female offspring of irradiated dams had significantly increased (15%) protein expression of PEPCK in the liver compared to the offspring of Sham-irradiated dams (Fig 1C). There was a non-significant 12% decrease in PEPCK protein expression between male Sham and male 1000 mGy treatment groups (Fig 1D).

**Fig 2:**
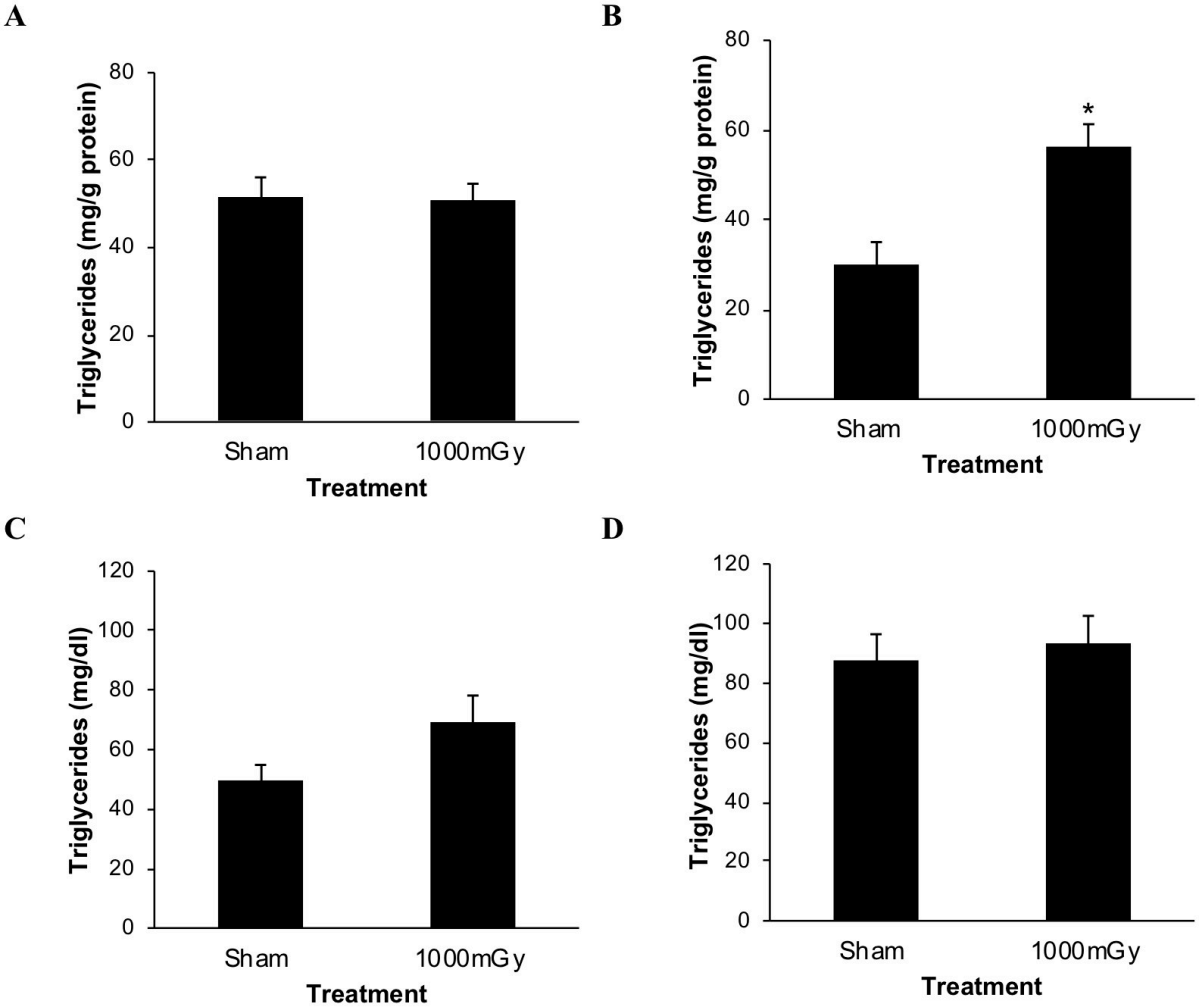
Triglyceride content in the liver and plasma from offspring of 1000 mGy irradiated and Sham-irradiated dams. (A) Female hepatic triglyceride content. (B) Male hepatic triglyceride content. (C) Female plasma triglyceride concentration. (D) Male plasma triglyceride concentration. Hepatic triglyceride content was normalized to liver protein content. Two-tailed t-test: Statistical significance between Sham and 1000 mGy groups is shown by * *p* ≤ 0.05. Data are presented as mean ± SEM. n = 7–10 per group.

A triglyceride assay was performed on liver tissue dissected from the mice at 4 months of age. No difference was observed in hepatic triglyceride content in the females based on treatment (Fig 2A). However, in the male offspring, the 1000 mGy treatment group exhibited higher hepatic triglyceride content than the Sham group (Fig 2B). Female plasma triglyceride concentration increased by 28% with treatment but the change was not significant (Fig 2C). No differences were observed from treatment in plasma triglyceride levels in the males (Fig 2D).

### Effect of 1000 mGy irradiation on glucose uptake in offspring IBAT

**Fig 3:**
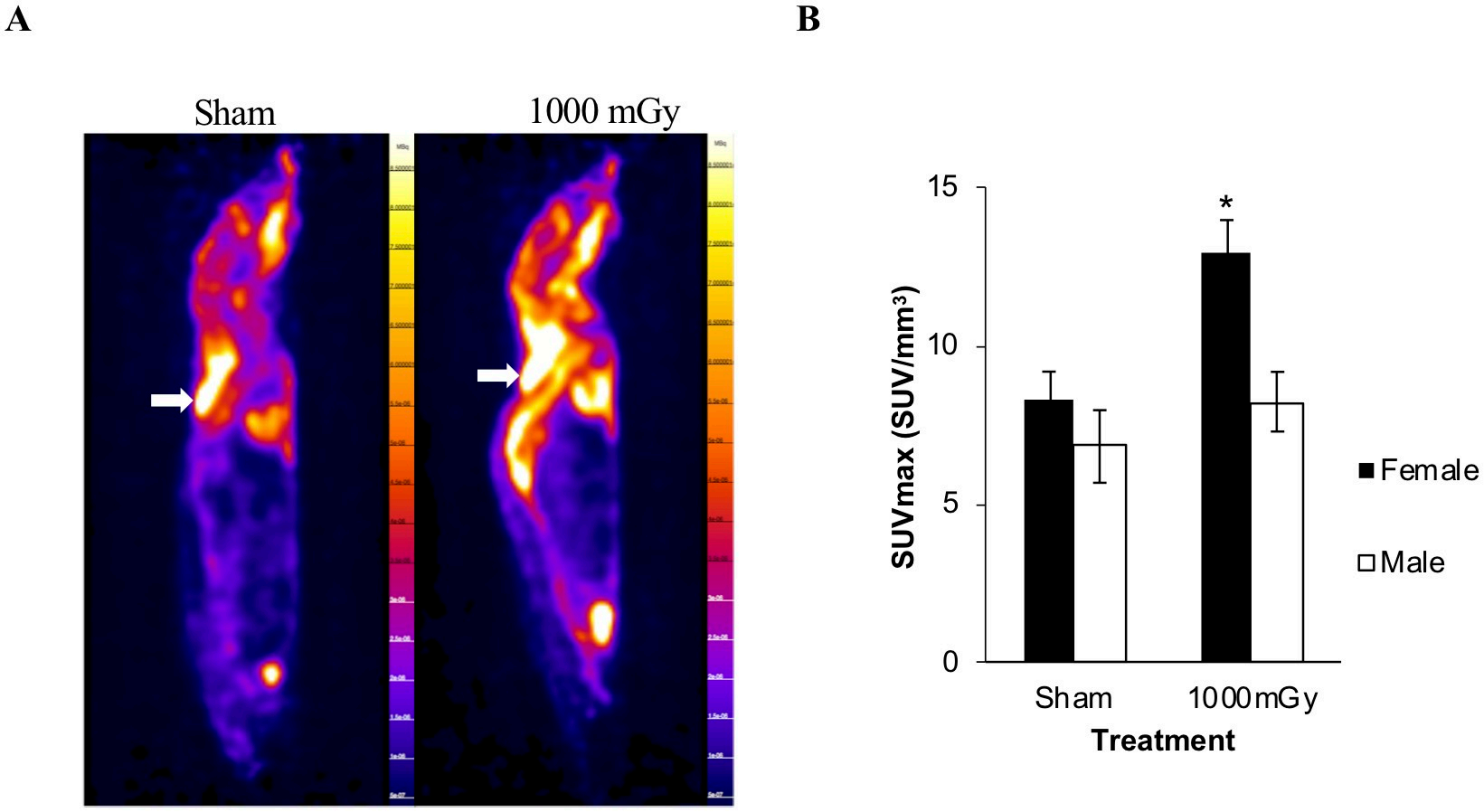
^18^F-FDG uptake. (A) Sagittal view of representative PET scans with ^18^F-FDG for Sham and 1000 mGy female mice. IBAT is indicated with the white arrows. (B) Black bars represent female IBAT ^18^F-FDG uptake. White bars represent male IBAT ^18^F-FDG uptake. Two-tailed t-test: Statistical significance between Sham and 1000 mGy groups is shown by * *p* ≤ 0.05. Data are presented as mean ± SEM. n = 7–10 per group.

Tissue specific glucose uptake was measured *in vivo* with a 10-minute static PET scan with ^18^F-FDG (Fig 3). Glucose uptake increased by 36% in female offspring of dams irradiated at 1000 mGy compared to female Sham offspring (Fig 3B). There was no significant change with treatment in the male offspring.

### IBAT signaling

**Fig 4:**
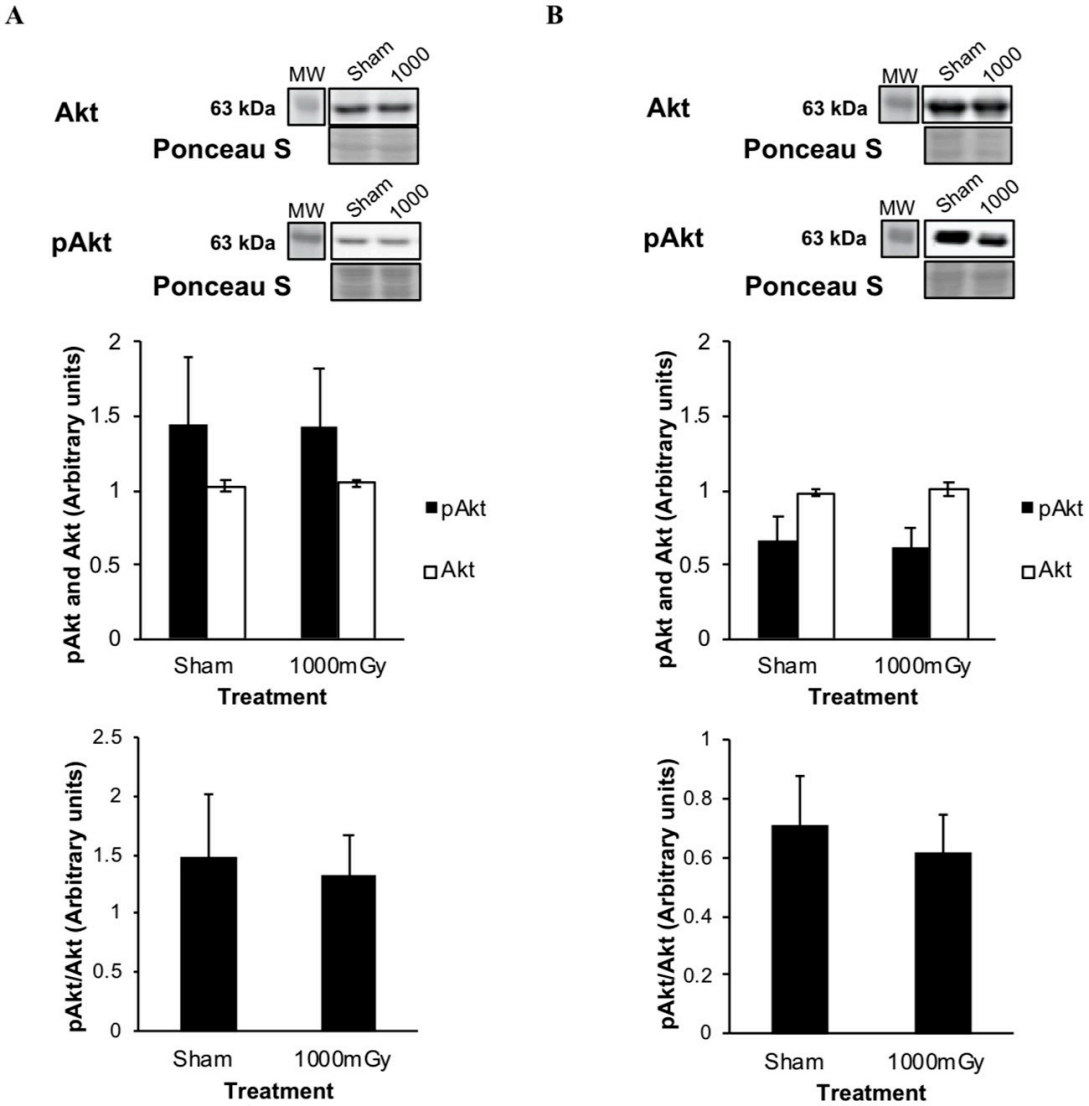
IBAT phosphorylated Akt (Ser473) (pAkt) and total Akt protein expression from offspring of 1000 mGy irradiated and Sham-irradiated dams. (A) Female pAkt and total Akt protein expression. (B) Male pAkt and total Akt protein expression. Black bars represent the phosphorylation of Akt. White bars represent total Akt protein expression. Below is the ratio of pAkt to total Akt. Ponceau S stains are shown as markers of equal protein loading. Results were normalized to loading controls. Two-tailed t-test: Statistical significance between Sham and 1000 mGy groups is shown by * *p* ≤ 0.05. Data are presented as mean ± SEM. n = 7–10.

The phosphorylation and expression of Akt, a signaling protein associated with insulin signaling was measured in IBAT. In female offspring, treatment did not result in significant differences in phosphorylated Akt Ser473 (pAkt) (*p* = 0.99) or total Akt (*p* = 0.70) (Fig 4A). When presented as the ratio of pAkt to total Akt, there remained no difference in the females. In male offspring, there are no significant changes in pAkt (*p* = 0.80) or total Akt (*p* = 0.60) (Fig 4B). Similarly, there were no significant differences observed from the ratio of pAkt to total Akt in male offspring between the treatment groups.

**Fig 5:**
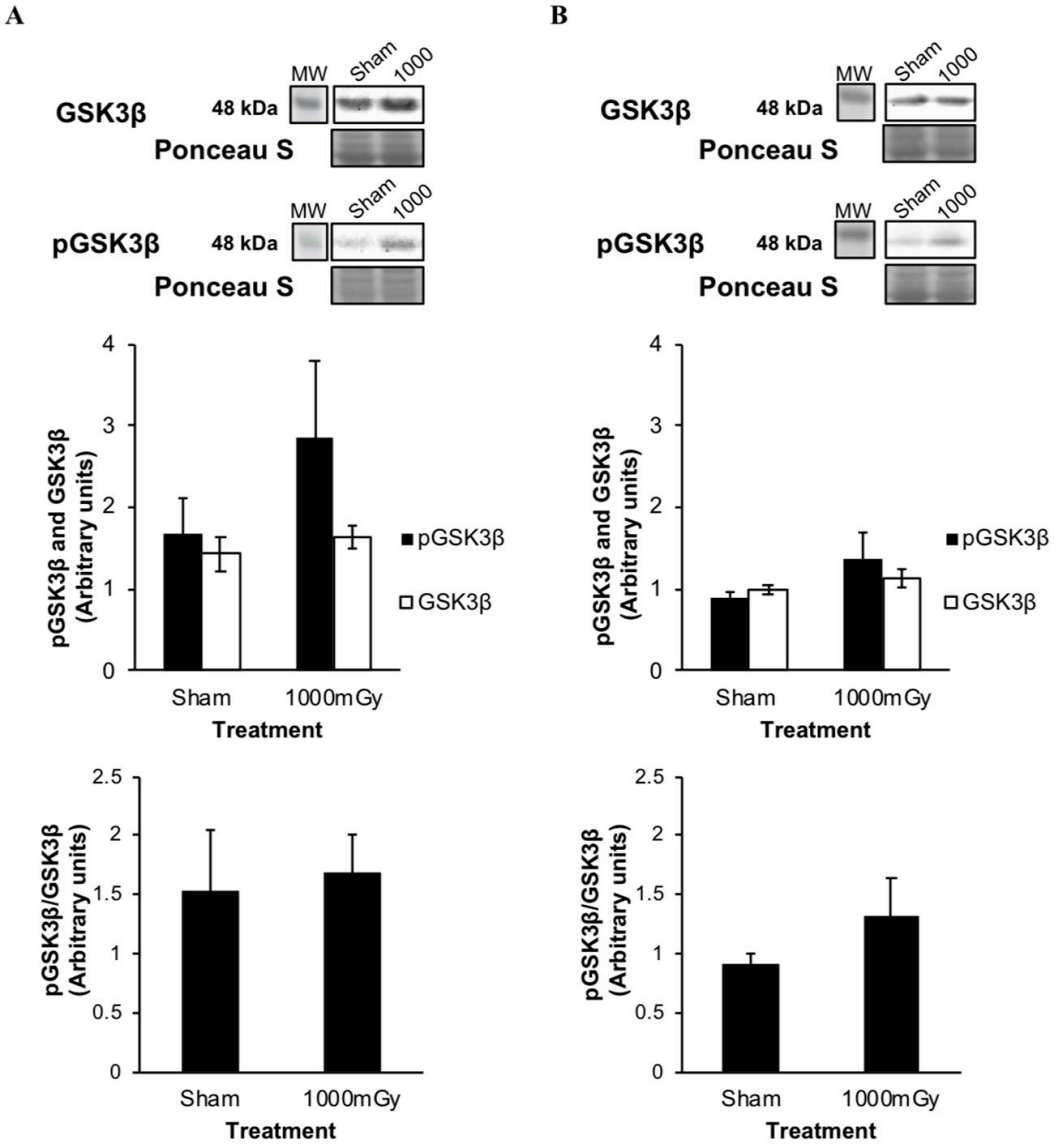
IBAT phosphorylated GSK3β Ser9 (pGSK3β) and total GSK3β protein expression from offspring of 1000 mGy irradiated and Sham-irradiated dams. (A) Female offspring pGSK3β and total GSK3β protein expression. Below is the ratio of pGSK3β to total GSK3β. (B) Male offspring pGSK3β and total GSK3β protein expression. Black bars represent the phosphorylation of GSK3β. White bars represent total GSK3β protein expression. Below is the ratio of pGSK3β to total GSK3β. Ponceau S stains are shown as markers of equal protein loading. Results were normalized to loading controls. Two-tailed t-test: Statistical significance between Sham and 1000 mGy groups is shown by * *p* ≤ 0.05. Data are presented as mean ± SEM. n = 7–10.

The phosphorylation and expression of GSK3β, a signaling protein associated with β-adrenergic signaling was measured in IBAT. There was no difference in total GSK3β protein expression in the female offspring between treatments, however the 1000 mGy offspring demonstrated a non-significant increase of 41% in the phosphorylation of Ser9 (pGSK3β) (*p* = 0.27) (Fig 5A). There was a 9% increase observed in the ratio of pGSK3β to total GSK3β in females with treatment when compared to Sham-irradiated (*p* = 0.82). In the males, there were non-significant increases of 14% (*p* = 0.31) in total GSK3β protein expression, 36% (*p* = 0.22) in pGSK3β (Fig 5B), and of 31% for the ratio of pGSK3β to total GSK3β (*p* = 0.36).

**Fig 6:**
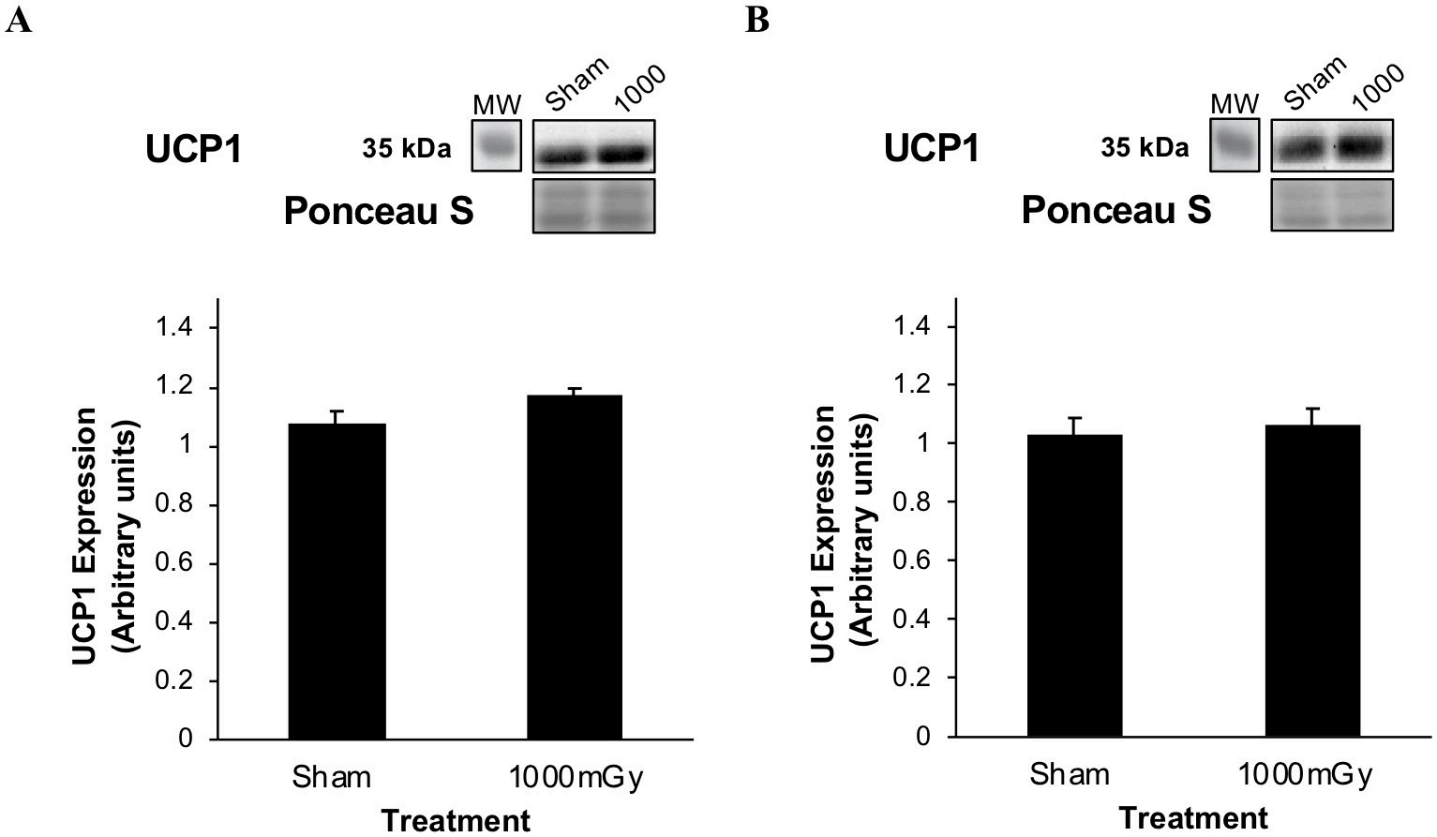
UCP1 protein expression from offspring of 1000 mGy irradiated and Sham-irradiated dams. (A) Female offspring IBAT UCP1 protein expression. (B) Male offspring IBAT UCP1 protein expression. Results were normalized to loading controls. Ponceau S stains are shown as markers of equal protein loading. Two-tailed t-test: Statistical significance between Sham and 1000 mGy groups is shown by * *p* ≤ 0.05. Data are presented as mean ± SEM. n = 7–10 per group.

UCP1 can be used as a marker of thermogenic activity of IBAT and is activated by β-adrenergic signaling. There was a non-significant increase of 9% in UCP1 protein expression between treatments in the females (*p* = 0.07) (Fig 6A). There was a non-significant increase of 3% in the males (*p* = 0.69) (Fig 6B).

## Discussion

While radiation protection regulations and standards are in place to prevent repeated and prolonged exposure, there are concerns about low dose exposures in humans. Even less is known about the effects of low dose radiation when exposed *in utero*. Radiation exposure during pregnancy presents a stress event that has the potential to permanently alter fetal metabolic processes. The effects may not be observable at birth or in early life but adult disease may arise at a younger age than expected. To our knowledge, we are the first to investigate alterations in physiology and metabolism due to fetal programming in a mouse model using low to SLDR. The data demonstrate that fetal programming can be caused by SLDR in mice altering liver gene and protein expression of targets associated with insulin resistance and alterations in IBAT glucose uptake. These findings may have important implications for estimating the effects of radiation on fetal programming in humans.

### Metabolic alterations in the liver

The increased SOCS3 and PEPCK gene expression and increased liver weight in female offspring led us to continue to examine the liver for signs of metabolic alterations. Targets of insulin resistance were measured in both sexes using protein expression from western blots. SOCS3, a known modulator of insulin resistance in the liver, is a protein induced by proinflammatory cytokines that directly inhibits IRS-1/IRS-2 by binding to specific sites, inhibiting phosphorylation and targeting the receptor substrates for degradation [48, 49]. In turn, this causes decreased activity in downstream components of the insulin signaling pathway. Therefore, SOCS3 is important in mediating insulin stimulated glucose uptake. Insulin resistance is a common consequence of exposure to stress, where stress results in the activation of proinflammatory cytokines that will upregulate SOCS3 [50]. In models of insulin resistance, SOCS3 protein expression is elevated in the liver [49, 51]. From our results, female SOCS3 expression increased in offspring of dams that were exposed to 1000 mGy of radiation (Fig 1A) suggesting the presence, or perhaps an early indicator, of insulin resistance.

PEPCK was used as a second indicator of insulin resistance in the liver. PEPCK is a rate-limiting enzyme that catalyzes the first step in gluconeogenesis, the conversion of oxaloacetate to phosphoenolpyruvate, and links glucose metabolism to the citric acid cycle [52]. In the liver, insulin signaling regulates gluconeogenesis by inhibiting key enzymes like PEPCK resulting in reduced hepatic glucose output. PEPCK is important in maintaining normal blood glucose levels [53]. Overexpression of PEPCK leads to insulin resistance in the liver [54, 55]. Previous work on models of fetal programming, specifically glucocorticoid exposure models, have shown increases in liver PEPCK mRNA expression and activity which are linked to glucose intolerance [55, 56]. The results of the present study demonstrate increased PEPCK protein expression in females that were exposed to radiation *in utero* (Fig 1C) which suggests insulin resistance. Increased PEPCK also suggests gluconeogenesis would be increased in female offspring. We are the first to show alterations in liver protein expression of PEPCK in a radiation model of fetal programming. With both targets of insulin sensitivity demonstrating increased expression, it can be concluded that female offspring of dams whole-body irradiated with 1000 mGy are more likely to be insulin resistant or develop insulin resistance in later life compared to the Sham-irradiated group.

### Hepatic triglycerides

Increased liver weight was observed in the female offspring of irradiated dams (Table 1) without the accumulation of hepatic triglycerides (Fig 2A). Increased liver weight in the absence of hepatic triglyceride accumulation could be a result of increased glycogen content [57]. In the fasting period the mice could have used less glycogen from their liver. A fetal programming model of undernutrition observed decreased glycogen content and liver [58]. A maternal streptozotocin diabetes fetal programming model saw increased liver glycogen and liver weight in newborn pigs [59].

The male offspring of irradiated dams exhibited increased hepatic triglycerides (Fig 2). Increased hepatic triglycerides are usually seen in advanced disease like non-alcoholic fatty liver disease; however this was not a model of a diseased state. Lipid accumulation in the liver is normally associated with increased circulating levels. However, in our model we fasted the mice for 5 hours prior to blood collection this could have resulted in circulating triglyceride levels decreasing and could have masked any differences between the groups [60]. Even in group with NAFLD fasting results in lowered circulating triglyceride levels. FASN (fatty acid synthase) gene expression can be used to explain these results since its expression is inversely based on triglyceride levels. When triglyceride levels are elevated, the formation of its precursor fatty acid by FASN would need to be reduced. Therefore, since only males showed decreased FASN expression to prenatal 1000 mGy compared to Sham, we predict that these livers are probably attempting to reduce fatty acid production to compensate for the increased triglyceride levels.

### IBAT glucose uptake and signaling

Tissue specific glucose uptake was measured in IBAT with PET using ^18^F-FDG. We report here that female offspring of irradiated dams have increased ^18^F-FDG uptake in IBAT (Fig 3). This is a basal increase in activity of about 1.5-fold. This magnitude of change is similar to a study in humans that exposed men to one month of overnight cold exposure (19°C). In this study, they reported increased BAT activity of about 1.5-fold [61]. This suggests that female offspring have hyperactive IBAT that uses more glucose and has increased energy expenditure compared to Sham-irradiated offspring. While there was no significant change in the weight of the tissue, IBAT ^18^F-FDG uptake may have increased to compensate for whole-body glucose intolerance. Compensation like this was observed in a study by Dumortier et al. 2017, in a fetal programming model of maternal low protein diet, where insulin secretion was impaired yet mice were able to maintain normal blood glucose levels and normal insulin sensitivity [2]. They observed increased energy expenditure by indirect calorimetry and hypothesized that increased IBAT uptake acts as a protection mechanism from changes in energy homeostasis and can protect against high-fat diet induced obesity. The protective effect was maintained in mice at 10 months of age but not at 18 months.

To correlate with the increase in ^18^F-FDG uptake in females, it was hypothesized that plasma triglyceride concentration would decrease in the offspring from the treatment group. Decreased plasma triglycerides would indicate that BAT has increased activity and is using triglycerides for energy [62]. Unexpectedly, this was not the case. The opposite trend was observed where an increase in plasma triglycerides was seen in the females (Fig 2C).

Phosphorylation of both Akt and GSK3β would have indicated that glucose uptake in IBAT is stimulated by insulin signaling [63]. However, there was no change in total Akt expression or more importantly phosphorylated Akt (Figs 4 and 5). While there was no significant change in pGSK3β (Fig 5A), there was a trend to increased pGSK3β in the female offspring (41%) leaving open the possibility that β-adrenergic activity may be involved in the increased IBAT ^18^F-FDG uptake.

To further investigate the regulation of IBAT activation, we measured UCP1 expression in IBAT. The presence of UCP1 in females and males from both treatment groups confirm that it is indeed IBAT that we are testing since white adipose tissue does not contain this protein [64]. UCP1 activation and expression increases through the β-adrenergic pathway [65]. The increase in UCP1 expression in female offspring of irradiated dams (Fig 6A), while not significant (*p* = 0.07), in combination with the increase seen in phosphorylated GSK3β show a trend toward the β-adrenergic pathway being responsible for the increase in female IBAT uptake. This is not surprising since β-adrenergic signaling is responsible for majority of the activity in BAT [66–69].

### Sex-dependent fetal programming results

Differences between males and females seen in the results of this study are not surprising based on previous fetal programming literature that have found sex differences in cardiovascular and metabolic function [70, 71]. The changes seen may be a result of differences in hormone concentrations between males and females. Depending on the time point in the estrous cycle, protein expression in the liver and brown adipose may vary because of the changes in hormone levels. Shen and Shi 2015, review the different sex hormones and their roles in glucose and lipid homeostasis in the liver [72]. Fernandez-Perez et al. 2013, discuss responses of the liver to estrogen and growth hormone that result in sexual dimorphism [73]. Various hepatic genes are up or down-regulated by different patterns in GH and sex-steroids and can affect glucose and lipid metabolism [73]. There are still uncertainties about the roles of sex hormones and their underlying mechanisms in fetal programming.

The purpose of the present study was to measure early markers of fetal programming before the onset of chronic disease. Whole-body dysfunction in glucose metabolism comes later in life in other models of fetal programming. For example, glucose tolerance testing has been done in fetal programming models that are older than the mice used in this study [55, 74, 75]. Low protein models of fetal programming have observed that young offspring who are predisposed to diabetes with aging have reduced insulin secretion but only slight or no changes were observed in glucose intolerance [75, 76].

There is a paucity of knowledge on the effects of low to SLDR on fetal programming and the development of metabolic dysfunction. Radiation doses above 100 mGy are considered above low dose but are still sub-lethal in mice. There is evidence to suggest that SLDR in mice may be relevant to LDR exposure in humans. The effects on the offspring observed in this study from 1000 mGy whole-body irradiations on the dams, suggest that a one-time SLDR causes physiological changes in metabolic activity in the female liver based on increases in protein expression of targets of insulin resistance, SOCS3 and PEPCK. BAT ^18^F-FDG uptake was significantly increased in the female offspring of irradiated dams and it is possible that the increase was caused by an increase in β-adrenergic signaling. While our results demonstrate a trend towards elevated UCP1 expression BAT activity is largely regulated via β-adrenergic signaling [66–69]. Our results demonstrate a trend towards elevated UCP1 expression and signaling via the β-adrenergic pathway. Changes are significant at 4 months of age but may be exacerbated over time. Future research is needed to investigate alterations in older offspring (e.g. 6 months of age). This research supplements the need for better characterization of the effects of prenatal SLDR exposure in mice and low dose radiation in humans to assess risk during pregnancy.

## Supporting information

S1 Table RT-qPCR primer sequences. Forward and reverse primer sequences were designed using Primer-BLAST.
(PDF)

S2 Table Liver mRNA expression of *in utero* radiation exposed female offspring. Expression represented as fold change (± SEM) relative to Sham. ANOVA: statistical significance (*p* < 0.05) compared to Sham are represented as bolded red text (upregulated) or blue text (downregulated) (n = 7–8 per group).
(PDF)

S3 Table Liver mRNA expression of *in utero* radiation exposed male offspring. Expression represented as fold change (± SEM) relative to Sham. ANOVA: statistical significance (*p* < 0.05) compared to Sham are represented as bolded red text (upregulated) or blue text (downregulated) (n = 7–8 per group).
(PDF)

S1 Raw Images (PDF)
