# Supplementary material for: Dose threshold for radiation induced fetal programming in a mouse model at 4 months of age: Hepatic expression of genes and proteins involved in glucose metabolism and glucose uptake in brown adipose tissue
Source: PLoS One. 2020 Apr 21;15(4):e0231650. doi: 10.1371/journal.pone.0231650 (PMC7173787; doi:10.1371/journal.pone.0231650)
Supplement: S1 Table — Forward and reverse primer sequences were designed using Primer-BLAST. (PDF) [file pone.0231650.s001.pdf]

**S1 Table. RT-qPCR primer sequences.** Forward and reverse primer sequences were designed using Primer-BLAST.

| Gene Name | GENE ID | Sequence (5'→3') | | PCR Product Size (bp) | Annealing Temp. ($^0$C) |
|---|---|---|---|---|---|
| Liver X Receptor α | LXRa | Forward Primer: | AAGGGTGTCCTCACCTCCTC | 106 | 58 |
| | | Reverse Primer: | GAAGGAGCGCCTGTTACACT | | |
| Liver X Receptor β | LXRb | Forward Primer: | GTTGCTTCGAGCTACTCCCA | 90 | 58 |
| | | Reverse Primer: | GCGAGAGTTGCCTCTGTGTC | | |
| Sterol Regulatory Element Binding Protein-1c | SREBP-1c | Forward Primer: | CAGACTCACTGCTGCTGACA | 126 | 58 |
| | | Reverse Primer: | CTCCACTCACCAGGGTCTGC | | |
| Acetyl-CoA Carboxylase (ACC) | ACACA | Forward Primer: | CCTGAAGACCTTAAAGCCAATGC | 149 | 58 |
| | | Reverse Primer: | CCAGCCCACACTGCTTGTA | | |
| Fatty Acid Synthase (FAS) | FASN | Forward Primer: | TGCACCTCACAGGCATCAAT | 104 | 58 |
| | | Reverse Primer: | GTCCCACTTGATGTGAGGGG | | |
| Stearoyl-CoA Desaturase-1 | SCD1 | Forward Primer: | CAGGTTTCCAAGCGCAGTTC | 142 | 58 |
| | | Reverse Primer: | ACTGGAGATCTCTTGGAGCA | | |
| Solute Carrier Family 2 | GLUT2 | Forward Primer: | AGAGGAGACTGAAGGATCTGC | 134 | 58 |
| | | Reverse Primer: | TTGCTTTGATCCTTCCAAGTTTGT | | |
| Peroxisome Proliferative Activated Receptor, Gamma, Coactivator 1 Alpha | PPARGC1A | Forward Primer: | TGAAAAAGCTTGACTGGCGTC | 91 | 58 |
| | | Reverse Primer: | AGCAGCACACTCTATGTCACTC | | |
| Peroxisome Proliferative Activated Receptor, Gamma, Coactivator 1 Beta | PPARGC1B | Forward Primer: | CAGGGTGGGGACTCTGGA | 141 | 58 |
| | | Reverse Primer: | GGGCTCTGTCTCTGAGGTCT | | |
| Suppressor of Cytokine Signaling 3 | SOCS3 | Forward Primer: | GCGAGAAGATTCCGCTGGTA | 85 | 58 |
| | | Reverse Primer: | CCGTTGACAGTCTTCCGACA | | |
| Phosphoenolpyruvate Carboxykinase | PEPCK | Forward Primer: | GAACTGACAGACTCGCCCTA | 99 | 58 |
| | | Reverse Primer: | CTTGATGAACTCCCCATCTCCC | | |
| Insulin Receptor Substrate 1 | IRS1 | Forward Primer: | ACGAACACTTTGCCATTGCC | 87 | 58 |
| | | Reverse Primer: | CCTTTGCCCGATTATGCAGC | | |

| | | | | | |
|---|---|---|---|---|---|
| Glucokinase | GSK | Forward Primer: | TTGCAACACTCAGCCAGACA | 125 | 58 |
| | | Reverse Primer: | GGGCTCCCCTCCTTGTAGTA | | |
| Heat Shock Protein 5 | Grp78 | Forward Primer: | GTGTGTGAGACCAGAACCGT | 77 | 58 |
| | | Reverse Primer: | GCAGTCAGGCAGGAGTCTTA | | |
| Heat Shock Protein 90, beta (Grp94) | Grp94 | Forward Primer: | GACCTTCGGGTTCGTCAGAG | 83 | 58 |
| | | Reverse Primer: | AGCCTTCTCGGCTTTTACCC | | |
| Diacylglycerol O-acyltransferase 1 | DGAT1 | Forward Primer: | TTCCGTGTTTGCTCTGGCAT | 134 | 60 |
| | | Reverse Primer: | CAGCCCCACTGACCTTCTTC | | |
| Diacylglycerol O-acyltransferase 2 | DGAT2 | Forward Primer: | AACACGCCCAAGAAAGGTGG | 75 | 58 |
| | | Reverse Primer: | GTAGTCTCGGAAGTAGCGCC | | |
| β-actin | β-actin | Forward Primer: | GGCTGTATTCCCCTCCATCG | 154 | 58 |
| | | Reverse Primer: | CCAGTTGGTAACAATGCCATGT | | |
| Ribosomal Protein L29 | rpl29 | Forward Primer: | ACATGGCCAAGTCCAAGAAC | 175 | 58 |
| | | Reverse Primer: | TGCATCTTCTTCAGGCCTTT | | |
