# Supplementary material for: Dose threshold for radiation induced fetal programming in a mouse model at 4 months of age: Hepatic expression of genes and proteins involved in glucose metabolism and glucose uptake in brown adipose tissue
Source: PLoS One. 2020 Apr 21;15(4):e0231650. doi: 10.1371/journal.pone.0231650 (PMC7173787; doi:10.1371/journal.pone.0231650)
Supplement: S2 Table — Expression represented as fold change (± SEM) relative to Sham. ANOVA: statistical significance (p < 0.05) compared to Sham are represented as bolded red text (upregulated) or blue text (downregulated) (n = 7–8 per group). (PDF) [file pone.0231650.s002.pdf]

S2 Table. Liver mRNA expression of *in utero* radiation exposed female offpsring. Expression represented as fold change (± SEM) relative to Sham. Statistically significant ($p < 0.05$) changes compared to Sham are represented as bolded **red text** (upregulated) or **blue text** (downregulated) (n = 7 – 8 per group).

SYSTEM	GENE	5 mGy	10 mGy	50 mGy	300 mGy	1000 mGy
Glucose/ Insulin Signaling	PPARGC1A	0.98 ± 0.22	1.20 ± 0.24	0.93 ± 0.18	1.01 ± 0.23	**1.70 ± 0.34**
	PPARGC1B	0.99 ± 0.14	1.14 ± 0.20	0.91 ± 0.08	1.01 ± 0.21	**1.60 ± 0.22**
	SOCS3	0.82 ± 0.11	1.25 ± 0.18	1.15 ± 0.15	1.20 ± 0.39	**2.01 ± 0.40**
	PEPCK	1.50 ± 0.44	1.41 ± 0.19	1.62 ± 0.29	1.50 ± 0.27	**2.03 ± 0.39**
	IRS1	0.86 ± 0.12	0.81 ± 0.09	0.85 ± 0.08	0.73 ± 0.11	0.92 ± 0.07
	GSK	0.91 ± 0.15	0.97 ± 0.23	0.77 ± 0.10	0.76 ± 0.19	0.70 ± 0.11
	GLUT2	1.13 ± 0.08	0.98 ± 0.06	0.89 ± 0.05	1.07 ± 0.10	1.27 ± 0.08
Lipid Metabolism	LXRα	1.10 ± 0.06	0.95 ± 0.06	0.98 ± 0.07	0.88 ± 0.06	1.11 ± 0.06
	LXRβ	0.89 ± 0.10	0.91 ± 0.11	1.00 ± 0.07	1.00 ± 0.10	1.25 ± 0.08
	SREBP-1c	0.87 ± 0.17	0.80 ± 0.15	0.90 ± 0.17	0.78 ± 0.15	0.84 ± 0.18
	ACACA	0.89 ± 0.08	0.89 ± 0.10	0.84 ± 0.08	0.86 ± 0.11	1.02 ± 0.10
	FASN	0.93 ± 0.23	0.92 ± 0.19	0.73 ± 0.15	0.78 ± 0.19	0.99 ± 0.22
	SCD1	0.61 ± 0.08	0.68 ± 0.09	**0.48 ± 0.07**	**0.35 ± 0.05**	0.77 ± 0.12
