# Supplementary material for: Dose threshold for radiation induced fetal programming in a mouse model at 4 months of age: Hepatic expression of genes and proteins involved in glucose metabolism and glucose uptake in brown adipose tissue
Source: PLoS One. 2020 Apr 21;15(4):e0231650. doi: 10.1371/journal.pone.0231650 (PMC7173787; doi:10.1371/journal.pone.0231650)
Supplement: S3 Table — Expression represented as fold change (± SEM) relative to Sham. ANOVA: statistical significance (p < 0.05) compared to Sham are represented as bolded red text (upregulated) or blue text (downregulated) (n = 7–8 per group). (PDF) [file pone.0231650.s003.pdf]

S3 Table. Liver mRNA expression of *in utero* radiation exposed male offpsring. Expression represented as fold change (± SEM) relative to Sham. Statistically significant ($p < 0.05$) changes compared to Sham are represented as bolded **red text** (upregulated) or **blue text** (downregulated) (n = 7 – 8 per group).

SYSTEM	GENE	5 mGy	10 mGy	50 mGy	300 mGy	1000 mGy
Glucose/ Insulin Signaling	PPARGC1A	0.77 ± 0.12	0.61 ± 0.15	0.58 ± 0.17	0.93 ± 0.19	0.98 ± 0.23
	PPARGC1B	0.87 ± 0.10	1.00 ± 0.18	0.82 ± 0.11	1.20 ± 0.23	1.26 ± 0.22
	SOCS3	1.80 ± 0.47	1.22 ± 0.20	1.69 ± 0.26	**2.45 ± 0.47**	**2.06 ± 0.76**
	PEPCK	0.76 ± 0.18	0.75 ± 0.12	0.48 ± 0.10	0.78 ± 0.25	1.12 ± 0.16
	IRS1	1.02 ± 0.08	0.83 ± 0.08	1.17 ± 0.06	**1.53 ± 0.17**	1.10 ± 0.11
	GSK	0.77 ± 0.11	0.90 ± 0.08	1.30 ± 0.12	0.79 ± 0.12	0.89 ± 0.12
	GLUT2	1.10 ± 0.21	1.10 ± 0.22	1.16 ± 0.27	1.22 ± 0.26	1.30 ± 0.25
Lipid Metabolism	LXRα	0.99 ± 0.04	0.91 ± 0.07	1.09 ± 0.07	1.11 ± 0.05	1.14 ± 0.06
	LXRβ	1.15 ± 0.20	0.84 ± 0.24	1.22 ± 0.28	1.65 ± 0.44	1.63 ± 0.31
	SREBP-1c	0.93 ± 0.09	0.98 ± 0.14	0.95 ± 0.11	1.01 ± 0.10	0.78 ± 0.11
	ACACA	1.00 ± 0.15	1.00 ± 0.13	1.13 ± 0.13	1.22 ± 0.18	1.00 ± 0.14
	FASN	1.08 ± 0.28	0.82 ± 0.20	1.13 ± 0.48	1.09 ± 0.31	**0.54 ± 0.14**
	SCD1	1.01 ± 0.35	0.78 ± 0.27	1.11 ± 0.47	0.95 ± 0.35	0.90 ± 0.30
