# Supplementary material for: Dose threshold for radiation induced fetal programming in a mouse model at 4 months of age: Hepatic expression of genes and proteins involved in glucose metabolism and glucose uptake in brown adipose tissue
Source: PLoS One. 2020 Apr 21;15(4):e0231650. doi: 10.1371/journal.pone.0231650 (PMC7173787; doi:10.1371/journal.pone.0231650)

Supporting information

S1_raw_images

This supplement contains the original images of the Western blots and Ponceau S stains that were used to prepare Fig 1 and Fig 4-6.

Fig 1 panel A below shows the original Western blot for female offspring liver SOCS3. The lanes are numbered, labelled with each sample loaded, and indicate whether the sample is from the female offspring of a dam that was exposed to 1000 mGy of radiation (1000) or Sham irradiated (Sham). Lanes marked with an ‘X’ were not used in the main manuscript figure.

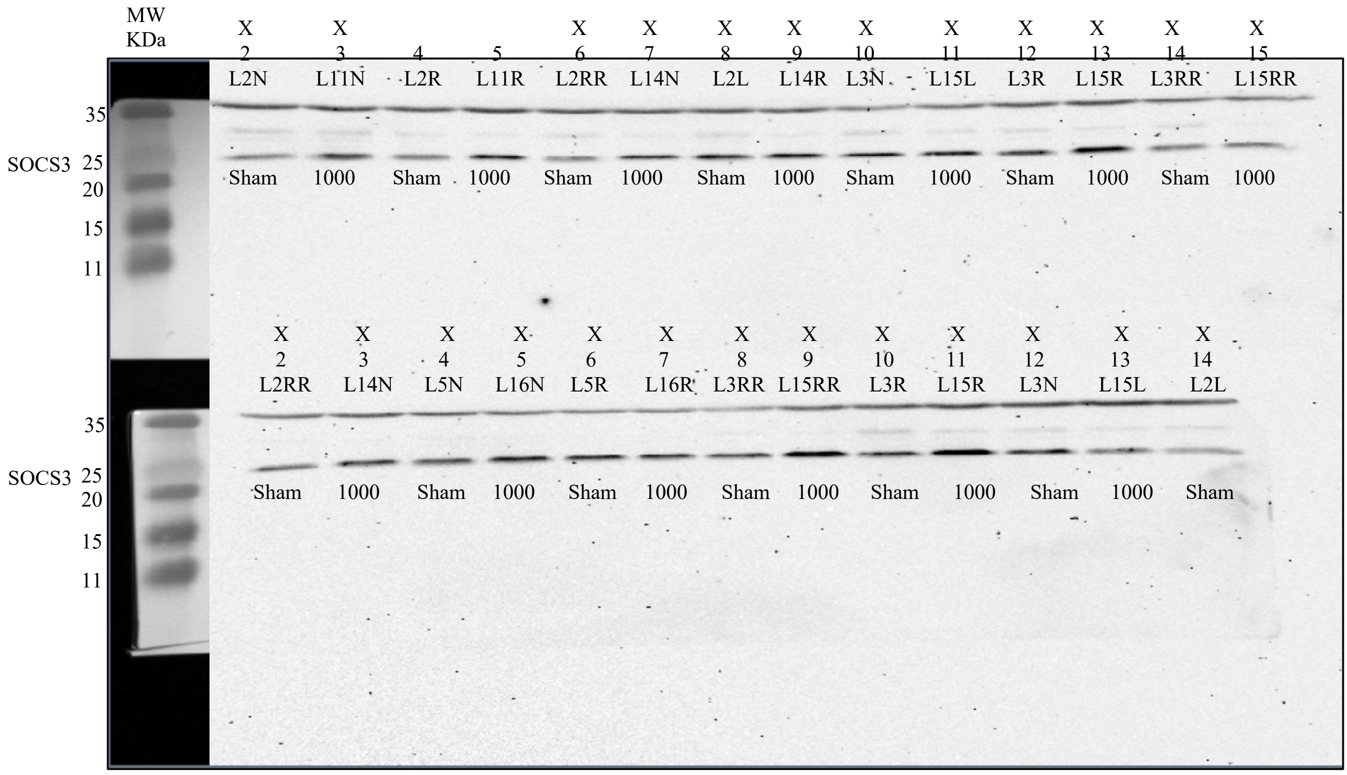

Fig 1 panel B below shows the original Western blot for male offspring liver SOCS3. The lanes are numbered, labelled with each sample loaded, and indicate whether the sample is from the male offspring of a dam that was exposed to 1000 mGy of radiation (1000), a Sham irradiated (Sham) dam or the female offspring of a Sham irradiated dam (FSham). Lanes marked with an 'X' were not used in the main manuscript figure.

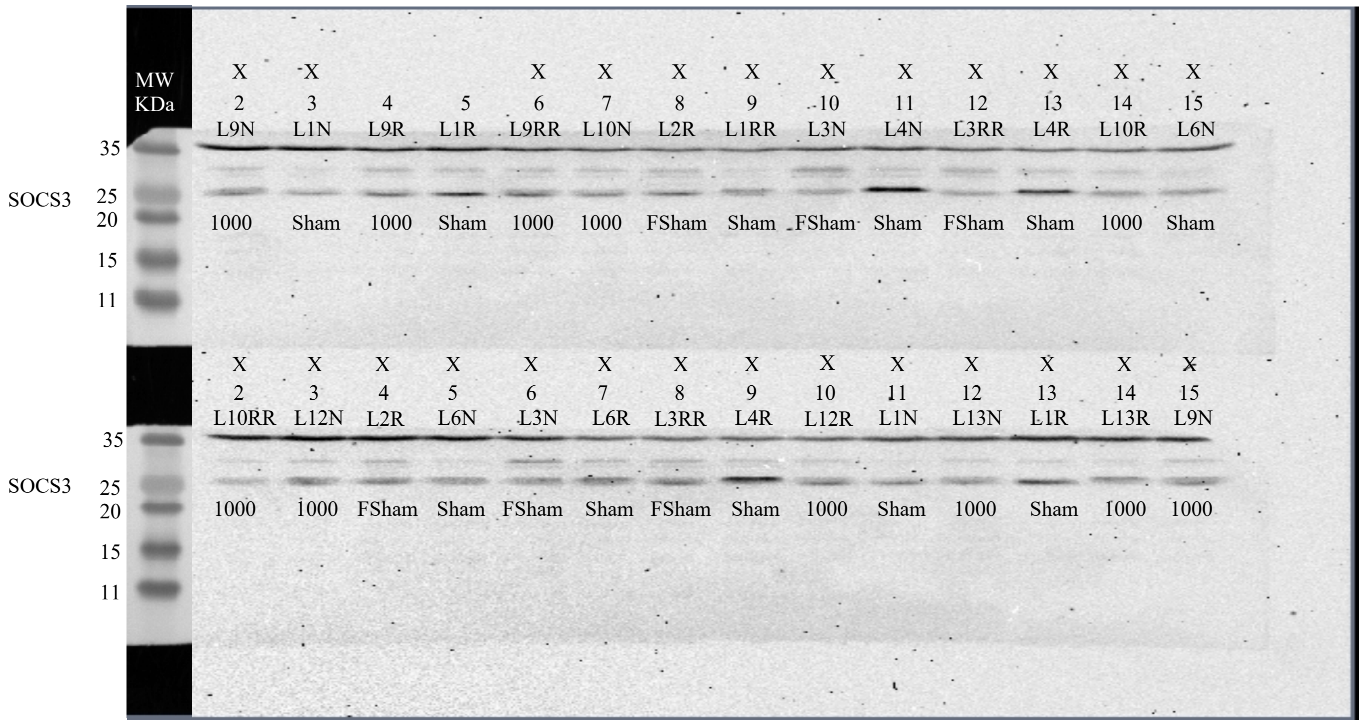

Fig 1 panel C below shows the original Western blot for female offspring liver PEPCK. The lanes are numbered, labelled with each sample loaded, and indicate whether the sample is from the female offspring of a dam that was exposed to 1000 mGy of radiation (1000) or Sham irradiated (Sham). Lanes marked with an ‘X’ were not used in the main manuscript figure.

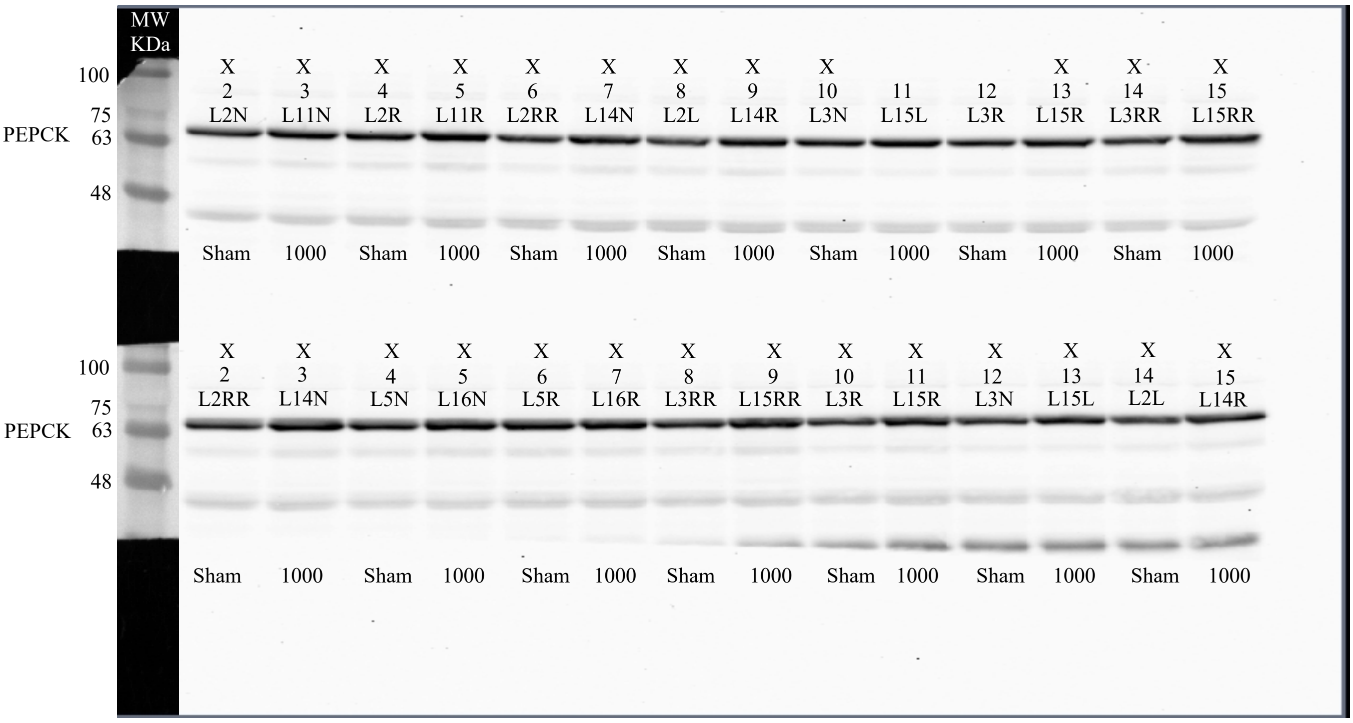

Fig 1 panel D below shows the original Western blot for male offspring liver PEPCK. The lanes are numbered, labelled with each sample loaded, and indicate whether the sample is from the male offspring of a dam that was exposed to 1000 mGy of radiation (1000), a Sham irradiated (Sham) dam or the female offspring of a Sham irradiated dam (FSham). Lanes marked with an 'X' were not used in the main manuscript figure.

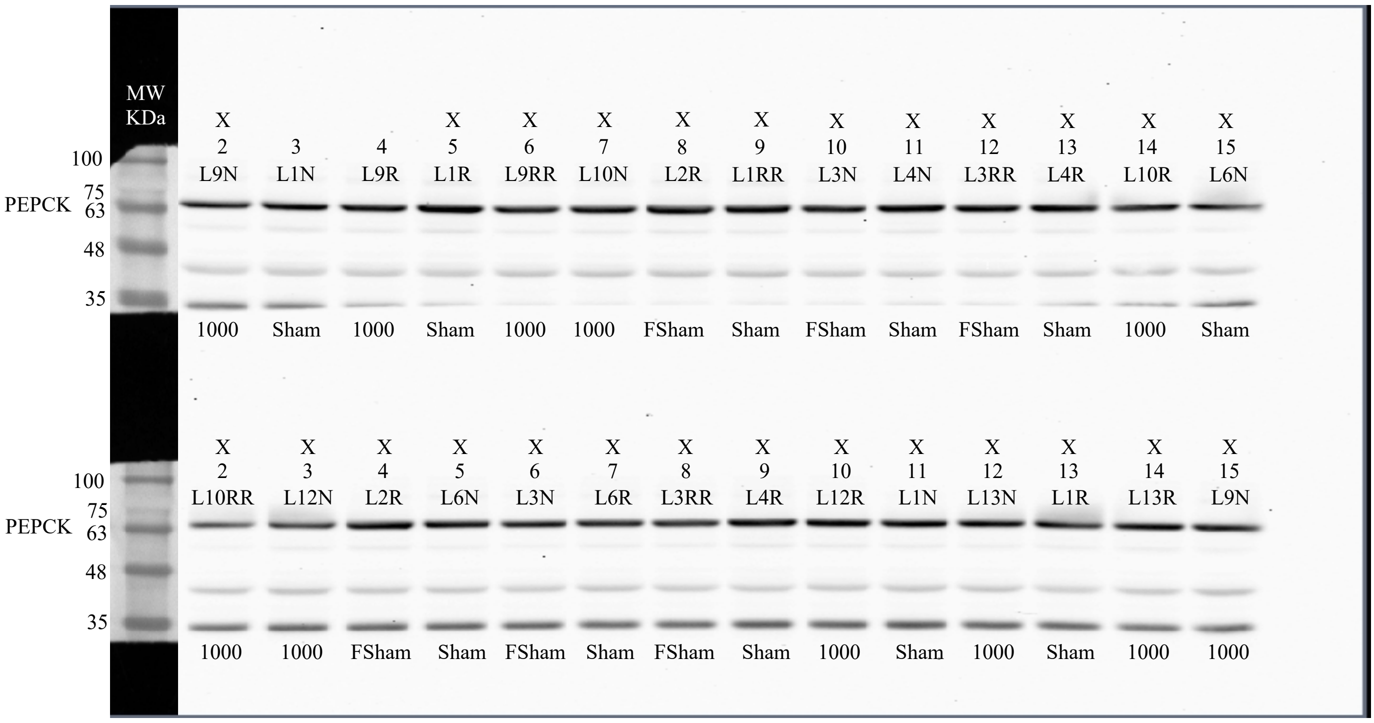

Fig 4 panel A below shows the original Western blot for female offspring IBAT Akt and p-Akt. The lanes are numbered, labelled with each sample loaded, and indicate whether the sample is from the female offspring of a dam that was exposed to 1000 mGy of radiation (1000), the female offspring of a Sham irradiated dam (Sham), or the male offspring of a dam that was exposed to 1000 mGy of radiation (M1000). Lanes marked with an 'X' were not used in the main manuscript figure.

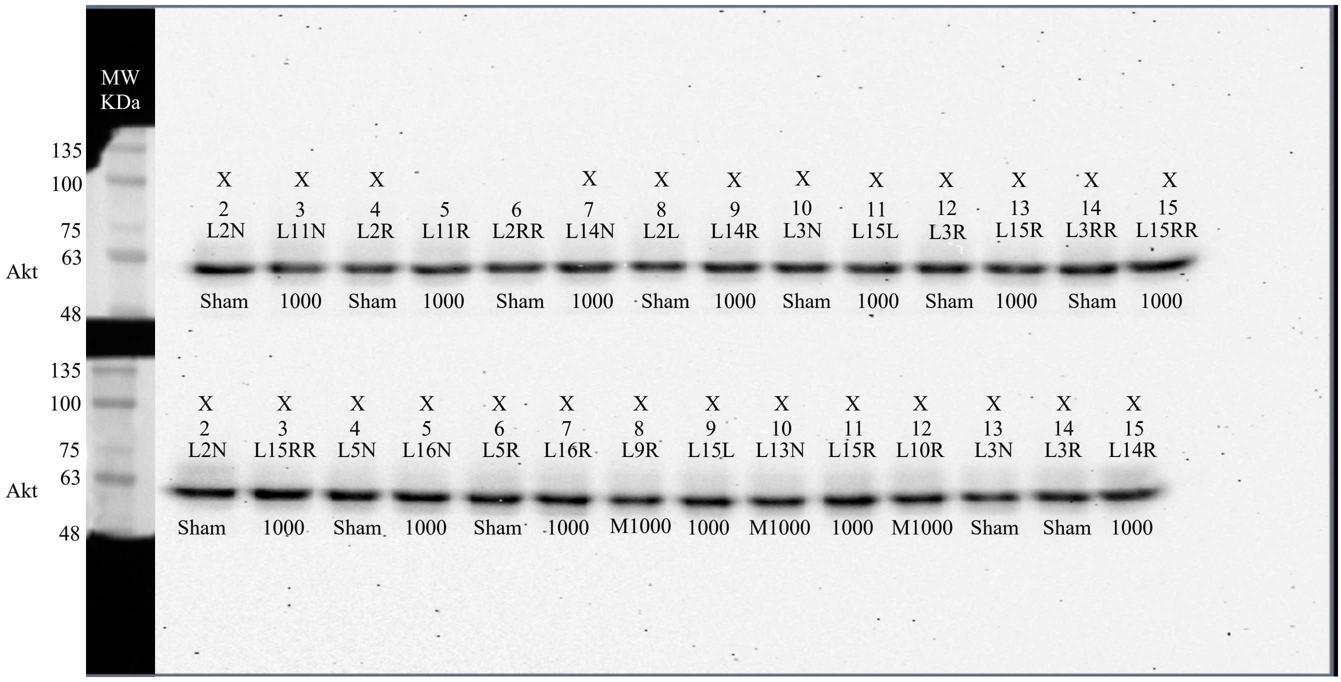

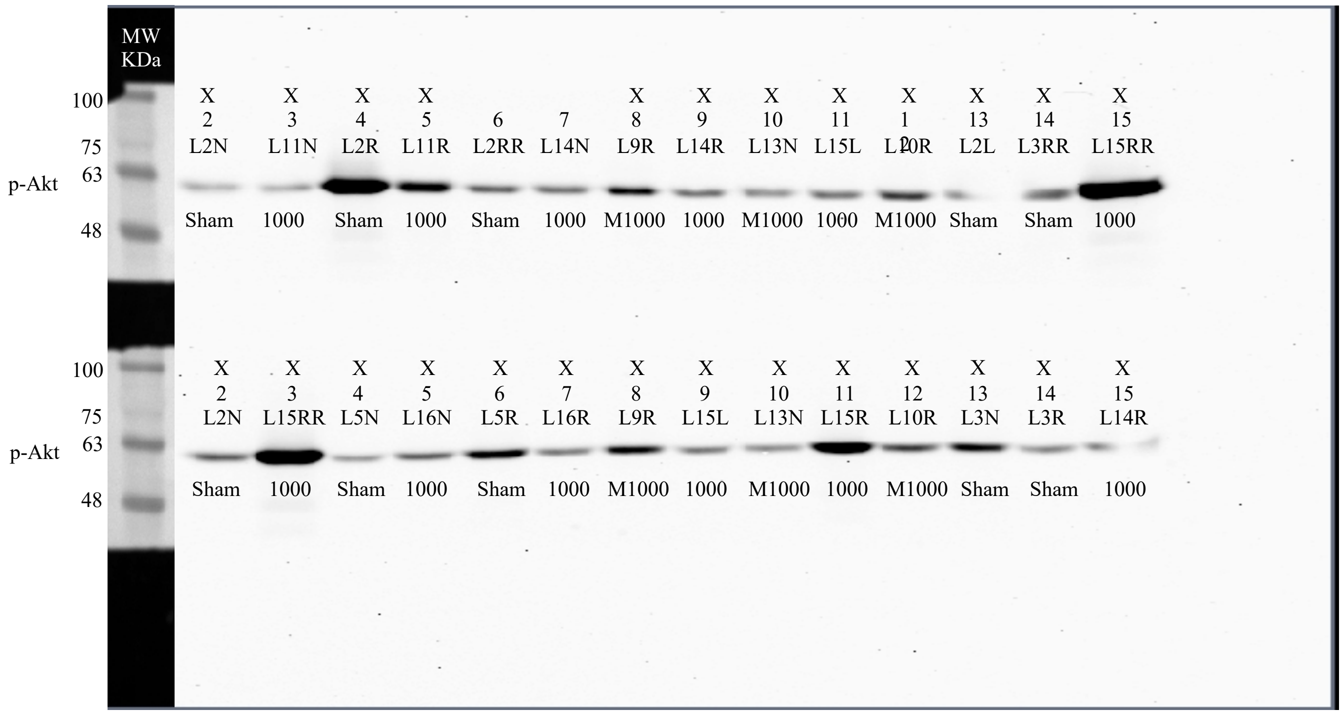

Fig 4 panel B below shows the original Western blot for male offspring IBAT Akt and p-Akt. The lanes are numbered, labelled with each sample loaded, and indicate whether the sample is from the male offspring of a dam that was exposed to 1000 mGy of radiation (1000), a Sham irradiated (Sham) dam or the female offspring of a Sham irradiated dam (FSham). Lanes marked with an 'X' were not used in the main manuscript figure.

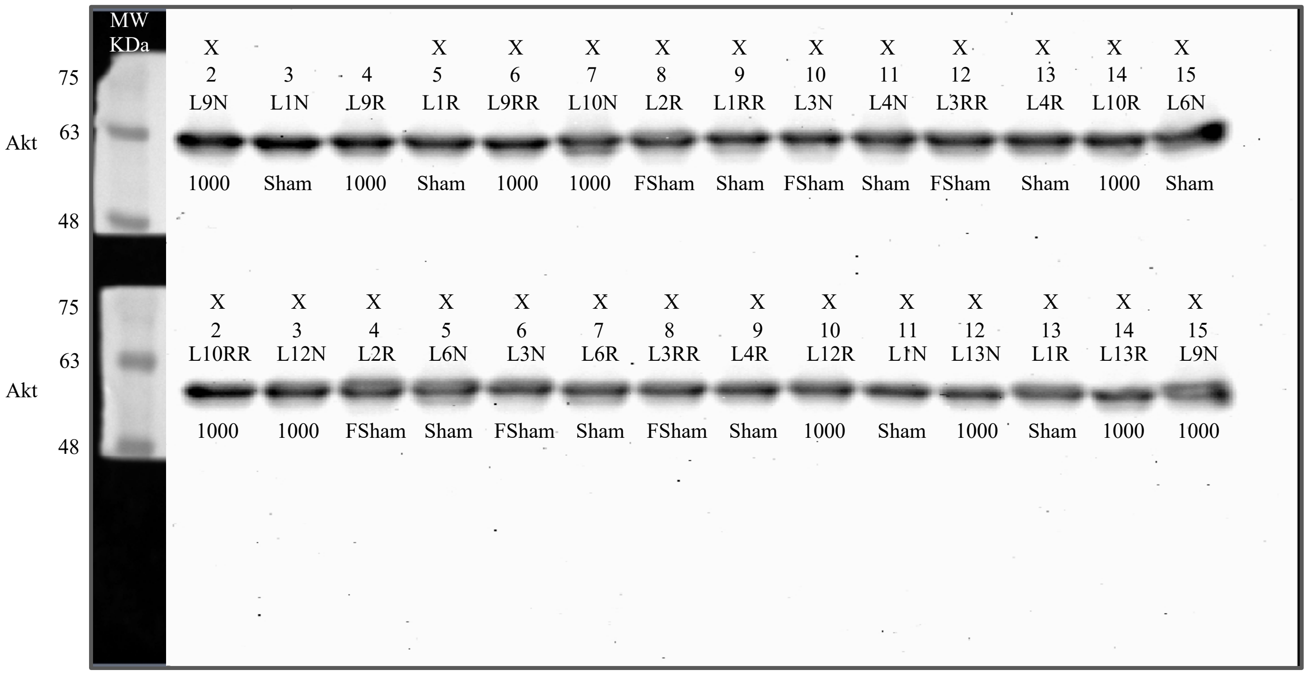

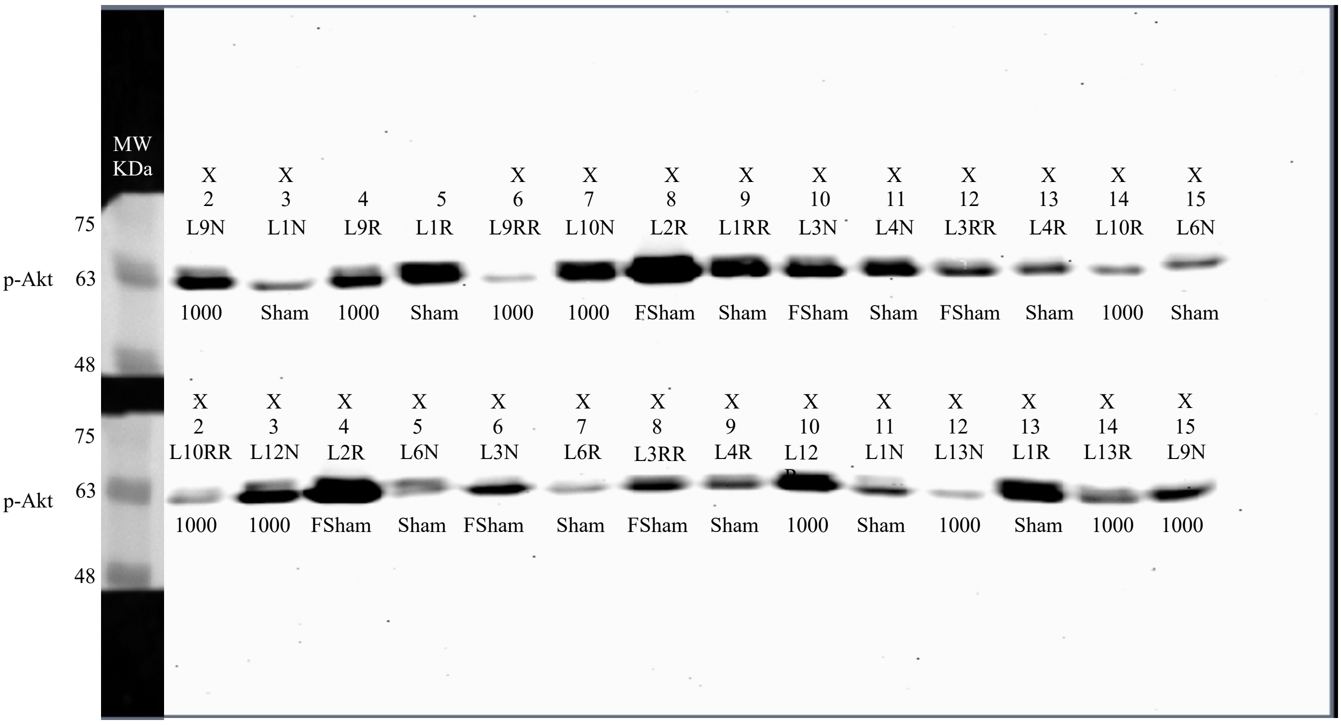

Fig 5 panel A below shows the original Western blot for female offspring IBAT GSK3β and p-GSK3β. The lanes are numbered, labelled with each sample loaded, and indicate whether the sample is from the female offspring of a dam that was exposed to 1000 mGy of radiation (1000), the female offspring of a Sham irradiated dam (Sham), or the male offspring of a dam that was exposed to 1000 mGy of radiation (M1000). Lanes marked with an 'X' were not used in the main manuscript figure.

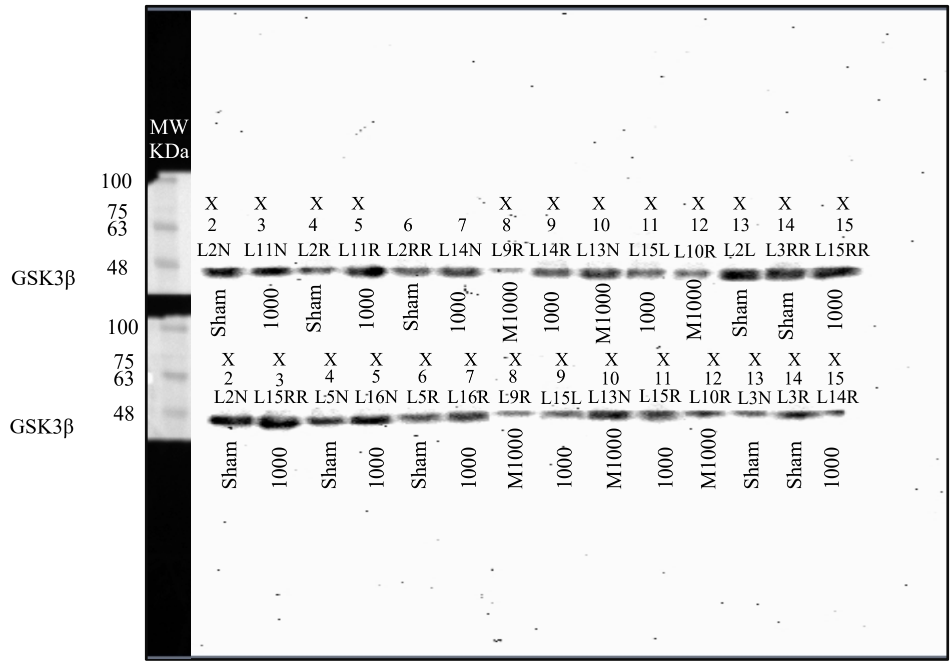

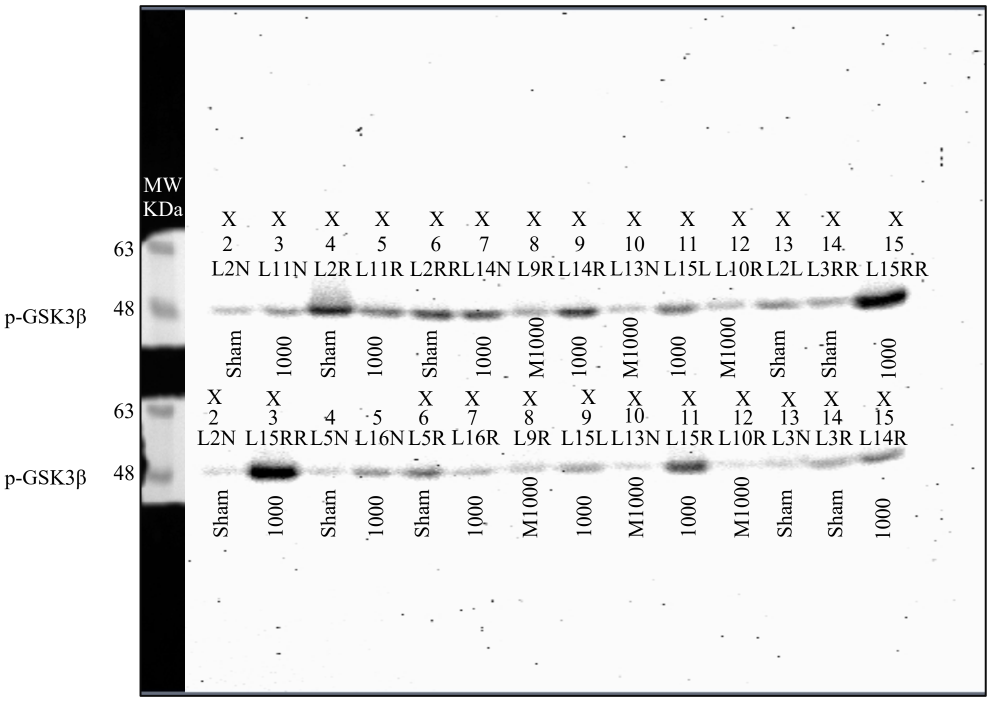

Fig 5 panel B below shows the original Western blot for male offspring IBAT GSK3β and p-GSK3β. The lanes are numbered, labelled with each sample loaded, and indicate whether the sample is from the male offspring of a dam that was exposed to 1000 mGy of radiation (1000), a Sham irradiated (Sham) dam or the female offspring of a Sham irradiated dam (FSham). Lanes marked with an 'X' were not used in the main manuscript figure.

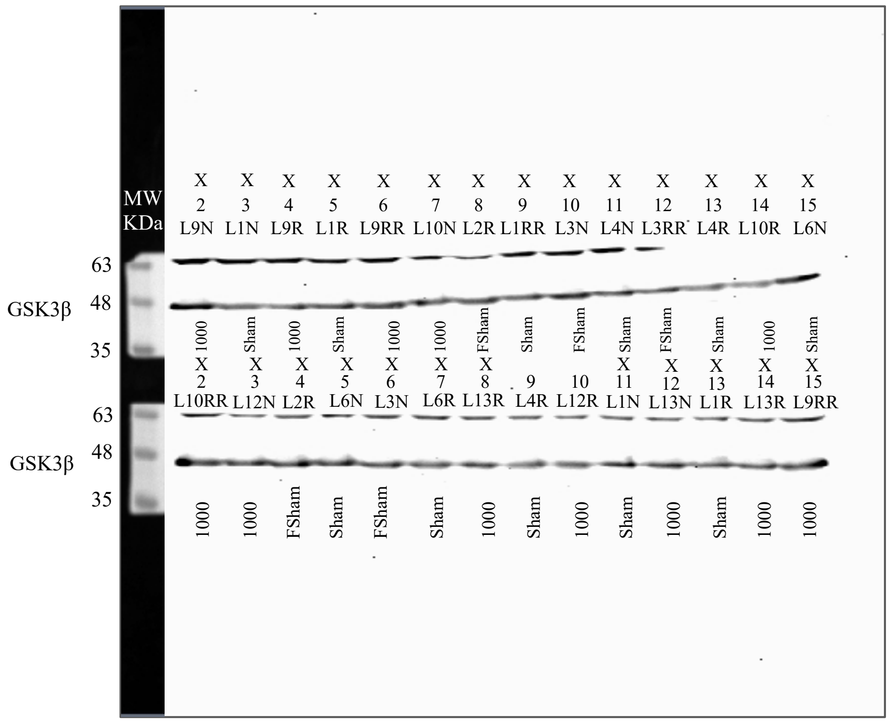

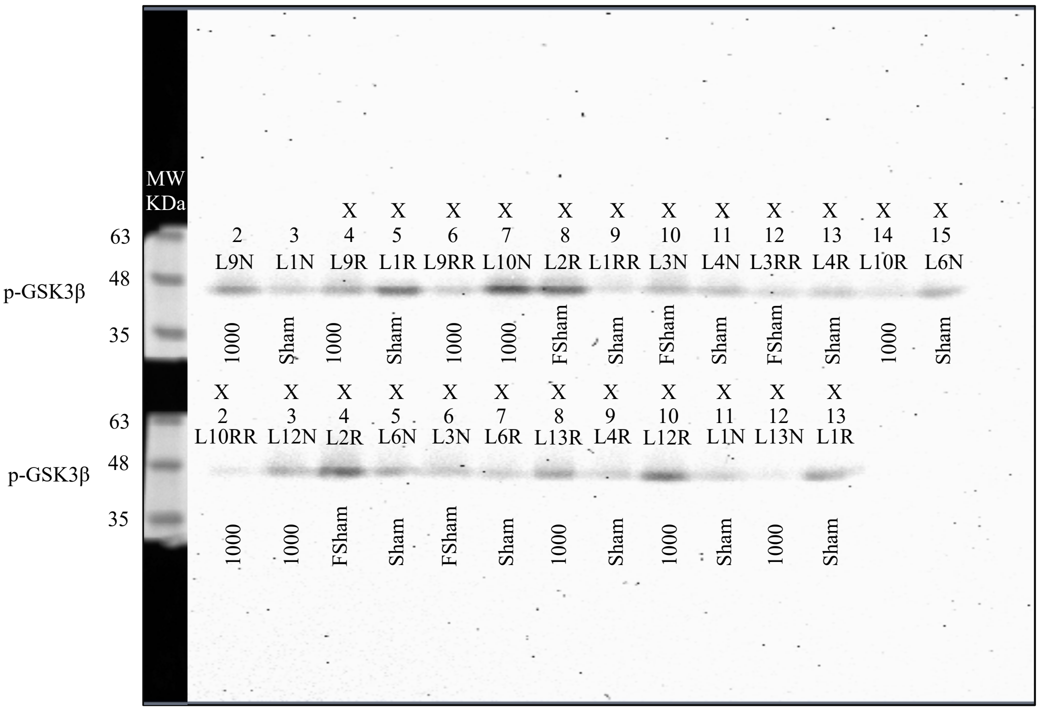

Fig 6 panel A below shows the original Western blot for female offspring IBAT UCP1. The lanes are numbered, labelled with each sample loaded, and indicate whether the sample is from the female offspring of a dam that was exposed to 1000 mGy of radiation (1000), the female offspring of a Sham irradiated dam (Sham), or the male offspring of a dam that was exposed to 1000 mGy of radiation (M1000). Lanes marked with an 'X' were not used in the main manuscript figure.

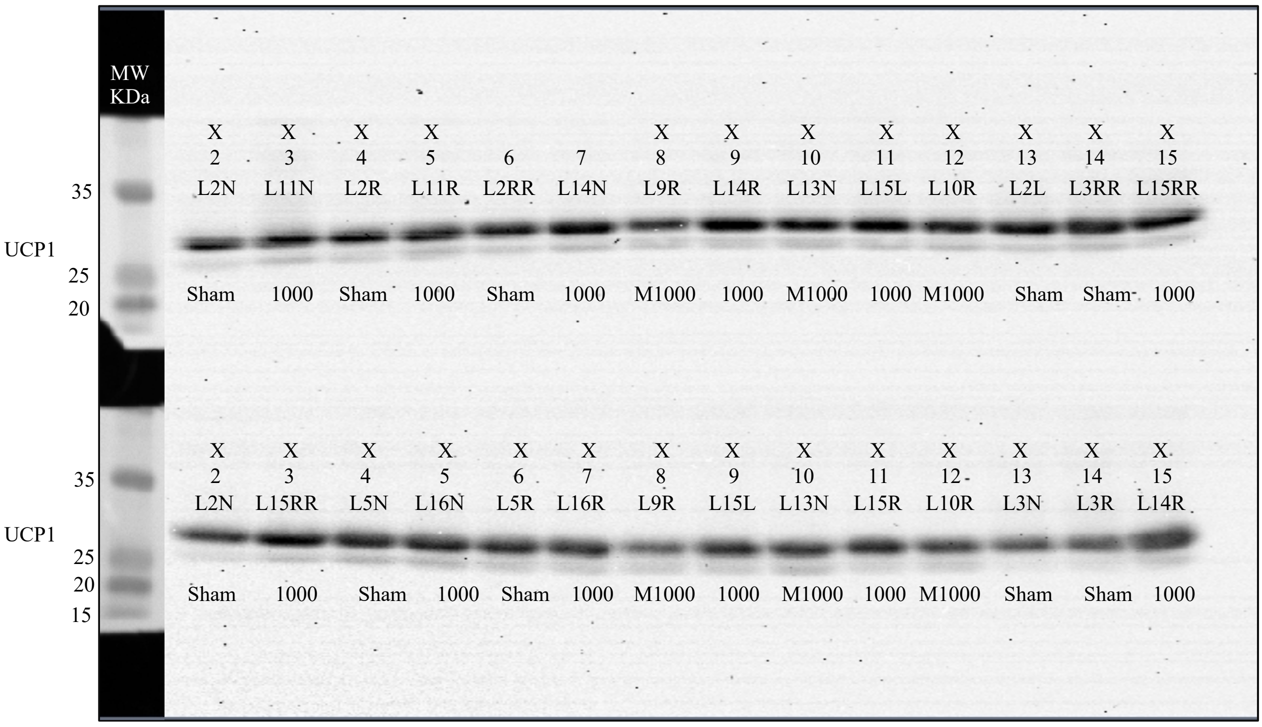

Fig 6 panel B below shows the original Western blot for male offspring IBAT UCP1. The lanes are numbered, labelled with each sample loaded, and indicate whether the sample is from the male offspring of a dam that was exposed to 1000 mGy of radiation (1000), a Sham irradiated (Sham) dam or the female offspring of a Sham irradiated dam (FSham). Lanes marked with an 'X' were not used in the main manuscript figure.

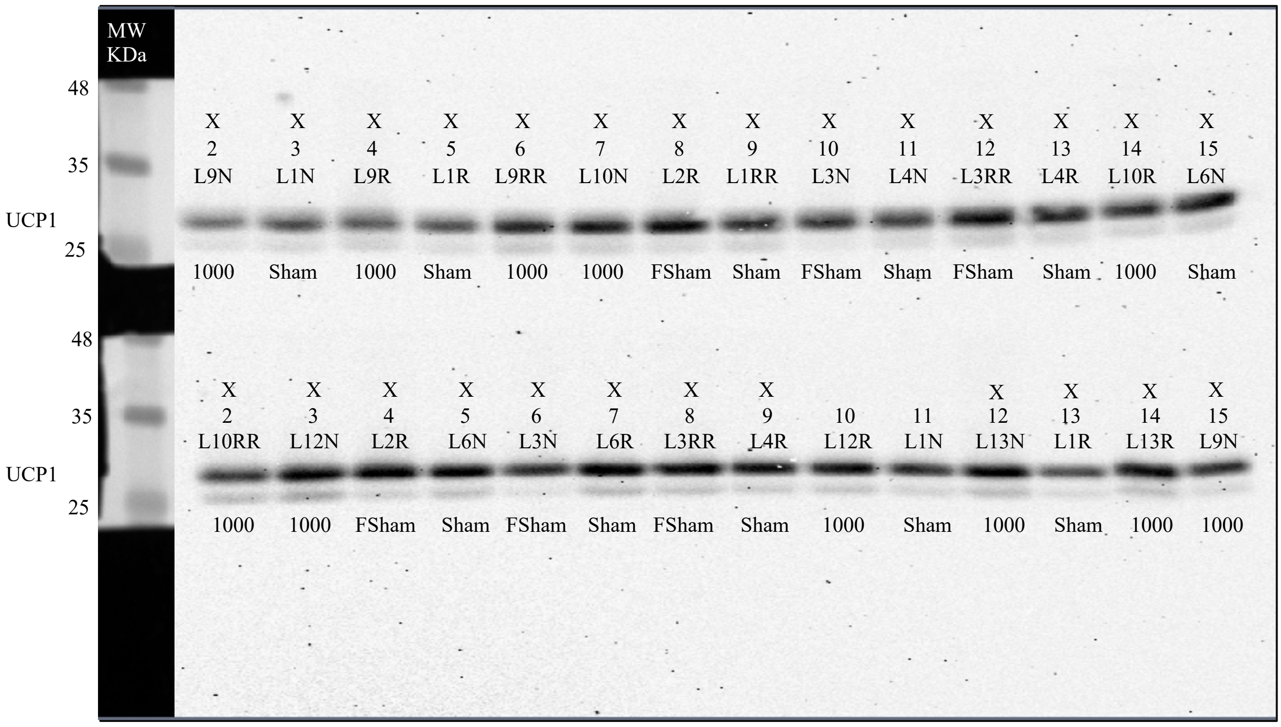

Supplement: S1 Raw Images — (PDF) [file pone.0231650.s004.pdf]
